# Network analysis of the progranulin-deficient mouse brain proteome reveals pathogenic mechanisms shared in human frontotemporal dementia caused by *GRN* mutations

**DOI:** 10.1186/s40478-020-01037-x

**Published:** 2020-10-07

**Authors:** Meixiang Huang, Erica Modeste, Eric Dammer, Paola Merino, Georgia Taylor, Duc M. Duong, Qiudong Deng, Christopher J. Holler, Marla Gearing, Dennis Dickson, Nicholas T. Seyfried, Thomas Kukar

**Affiliations:** 1 Department of Pharmacology and Chemical Biology, School of Medicine, Emory University, Atlanta, GA 30322 USA; 2 Center for Neurodegenerative Disease, School of Medicine, Emory University, Atlanta, GA 30322 USA; 3 Department of Neurology, Central South University, Second Xiangya Hospital, Changsha, 48105 Hunan China; 4 Department of Biochemistry, School of Medicine, Emory University, Atlanta, GA 30322 USA; 5 Department of Neurology, School of Medicine, Emory University, Atlanta, GA 30322 USA; 6 Department of Pathology and Laboratory Medicine, Emory University School of Medicine, Atlanta, GA 30322 USA; 7 Department of Neuroscience, Mayo Clinic, Jacksonville, FL USA

**Keywords:** Progranulin (PGRN), Frontotemporal lobar degeneration (FTLD), Frontotemporal dementia (FTD), Neurodegeneration, Lysosome, Inflammation, Proteomics, GPNMB, Galectin-3

## Abstract

Heterozygous, loss-of-function mutations in the granulin gene (*GRN*) encoding progranulin (PGRN) are a common cause of frontotemporal dementia (FTD). Homozygous *GRN* mutations cause neuronal ceroid lipofuscinosis-11 (CLN11), a lysosome storage disease. PGRN is a secreted glycoprotein that can be proteolytically cleaved into seven bioactive 6 kDa granulins. However, it is unclear how deficiency of PGRN and granulins causes neurodegeneration. To gain insight into the mechanisms of FTD pathogenesis, we utilized Tandem Mass Tag isobaric labeling mass spectrometry to perform an unbiased quantitative proteomic analysis of whole-brain tissue from wild type (*Grn*^+/+^) and *Grn* knockout (*Grn*^−/−^) mice at 3- and 19-months of age. At 3-months lysosomal proteins (i.e. Gns, Scarb2, Hexb) are selectively increased indicating lysosomal dysfunction is an early consequence of PGRN deficiency. Additionally, proteins involved in lipid metabolism (Acly, Apoc3, Asah1, Gpld1, Ppt1, and Naaa) are decreased; suggesting lysosomal degradation of lipids may be impaired in the *Grn*^−/−^ brain. Systems biology using weighted correlation network analysis (WGCNA) of the *Grn*^−/−^ brain proteome identified 26 modules of highly co-expressed proteins. Three modules strongly correlated to *Grn* deficiency and were enriched with lysosomal proteins (Gpnmb, CtsD, CtsZ, and Tpp1) and inflammatory proteins (Lgals3, GFAP, CD44, S100a, and C1qa). We find that lysosomal dysregulation is exacerbated with age in the *Grn*^−/−^ mouse brain leading to neuroinflammation, synaptic loss, and decreased markers of oligodendrocytes, myelin, and neurons. In particular, GPNMB and LGALS3 (galectin-3) were upregulated by microglia and elevated in FTD-*GRN* brain samples, indicating common pathogenic pathways are dysregulated in human FTD cases and *Grn*^−/−^ mice. GPNMB levels were significantly increased in the cerebrospinal fluid of FTD-*GRN* patients, but not in *MAPT* or *C9orf72* carriers, suggesting GPNMB could be a biomarker specific to FTD-*GRN* to monitor disease onset, progression, and drug response. Our findings support the idea that insufficiency of PGRN and granulins in humans causes neurodegeneration through lysosomal dysfunction, defects in autophagy, and neuroinflammation, which could be targeted to develop effective therapies.

## Background

Frontotemporal lobar degeneration (FTLD) is the most common cause of dementia in people under the age of 60 [7]. Frontotemporal dementia (FTD) is the clinical manifestation of FTLD neuropathology and major clinical symptoms can be divided into either progressive deficits in executive function and behavior or language [26]. Importantly, ~ 30% of FTD patients have a familial history of FTD or related neurodegenerative disease, highlighting the important role of genetics in disease pathogenesis [26, 39, 43]. Taken together autosomal dominant mutations in three genes, progranulin (*GRN*), chromosome 9 open reading frame 72 (*C9orf72*), and microtubule associated protein tau (*MAPT*), account for the majority of FTD heritability [43]. Less common mutations in other genes encoding TAR DNA binding protein 43 (TDP-43; *TARDBP*) [19, 32, 95], sequestosome-1/p62 (*SQSTM1*) [67, 121], charged multi-vesicular body protein 2b (*CHMP2B*) [104, 120], valosin-containing protein (*VCP*) [45, 126], TANK-binding kinase 1 (*TBK1*) [33, 38, 92], among other rare genes can cause FTD [44]. Despite these advances in understanding the genetic causes of FTD, the normal, physiologic function of many proteins encoded by genes mutated in FTD is still unclear. Understanding the function and dysfunction of proteins linked to FTD is critical to developing effective therapies.

In particular, the function of the progranulin (PGRN) protein, encoded by *GRN*, has remained an enigma. The PGRN protein family is over a billion years old and evolutionarily conserved across many species, suggesting PGRN has a critical function [85]. Heterozygous *GRN* mutations cause FTD by decreasing PGRN mRNA and protein by 50% or more [6, 24, 31, 36, 57, 73]. Although FTD-*GRN* mutations lead to TDP-43 pathology and dysfunction [17, 23, 81], it is still unclear why deficiency of PGRN ultimately causes neurodegeneration.

PGRN is a pleiotropic, cysteine rich secreted protein composed of one half-length and seven full-length domain repeats called granulins that are connected by peptide linker regions [112]. PGRN is highly expressed in neuronal and microglial cells throughout the brain [88]. PGRN has been implicated in various physiological functions, ranging from extracellular signaling through membrane receptors [25, 76, 80, 129], neuroprotection [35, 65, 117, 130], to modulating inflammation [21, 70, 137]. PGRN can be cleaved by a variety of proteases to release individual ~ 6-kDa granulins, which are also bioactive [8, 47, 137]. However, the precise functions of PGRN and granulins are unclear, and the pathways that lead from deficiency of PGRN and granulins to FTD are still unknown, which has impeded progress towards developing therapies for FTD-*GRN*.

Another roadblock in the development of therapies for FTD is a lack of specific and sensitive biomarkers [40]. The identification of reliable biomarkers is important to distinguish FTD from Alzheimer's disease or other neurodegenerative diseases. Importantly, appropriate biomarkers are also useful to monitor disease progression and assess efficacy of potential drugs. Elevated levels of neurofilament light chain (Nfl) in CSF and plasma are one promising biomarker for symptomatic FTD patients that harbor mutations in *GRN*, *C9orf72*, or *MAPT* [58]. However, increased levels of Nfl in plasma or CSF are not specific to FTD and Nfl is increased in several other neurodegenerative diseases [124]. More research is needed to identify novel biomarkers to diagnose, discriminate, and ultimately treat the different sub-types of FTD.

To investigate the function of PGRN, provide insight into FTD pathogenesis, and identify potential biomarkers, we performed an unbiased quantitative proteomics analysis of whole-brain tissue from wild type (*Grn*^+*/*+^) and *Grn* knockout (*Grn*^−/−^) mice at 3- and 19-months of age. We utilized the *Grn*^−/−^ mouse model because they share many pathological features with human FTD-*GRN*, including microgliosis, lipofuscinosis, accumulation of ubiquitinated proteins, and behavioral impairment [1, 82, 96]. We identified a variety of proteins that were increased or decreased in the *Grn*^−/−^ mouse brain proteome that were subsequently validated using biochemical and immunohistological approaches. In particular, lysosomal proteins were the most significantly altered in young *Grn*^−/−^ mice, indicating that lysosomal dysfunction is an early event when PGRN expression is lost. In addition, we identified two proteins, transmembrane glycoprotein NMB (GPNMB) and galectin-3, which are predominantly expressed in microglia and are key hubs of a larger network of dysregulated proteins that change with age in *Grn*^−/−^ mice. Moreover, GPNMB and galectin-3 were significantly elevated in the lysates of FTD-*GRN* brain samples compared to healthy controls. Together, these data suggest that common pathogenic pathways are dysregulated in both *Grn*^−/−^ mice and human FTD cases caused by *GRN* haploinsufficiency.

## Methods

### Mouse brain sample processing for proteomics and biochemical analysis

*Grn*^−/−^ mice used in this study were originally generated in Dr. Aihao Ding’s laboratory [131, 132] and purchased from the Jackson Laboratory (B6(Cg)-Grntm1.1Aidi/, IMSR Cat# JAX:013175, RRID:IMSR_JAX:013175). Mice were housed in the Department of Animal Resources at Emory University and all work was approved by the Institutional Animal Care and Use Committee (IACUC) and performed in accordance with the Guide for the Care and Use of Laboratory Animals of the National Institutes of Health. Mice were sacrificed at various ages and whole brains were dissected from the skull and frozen immediately in liquid nitrogen. Brain tissue was ground to a fine powder under liquid nitrogen using a mortar and pestle and stored at − 80 °C as previously described [48]. Frozen cerebral cortex brain samples from 3-month old *Grn*^+*/*+^ wild type (n = 4) and *Grn*^−/−^ knock out (n = 4) mice and 19-month old *Grn*^+*/*+^ wild type (n = 4) and *Grn*^−/−^ knock out (n = 4) mice were collected. For proteomic analysis, approximately 100 mg of mouse brain tissue powder was homogenized in 500 μL of urea lysis buffer (8 M urea, 100 mM NaH_2_PO_4_, pH 8.5), supplemented with 5 μL (100 × stock) HALT protease and phosphatase inhibitor cocktail (Pierce) using a Bullet Blender (Next Advance) and 750 mg of steel beads (Next Advance). Protein supernatants were then transferred to a new 1.5 mL Eppendorf tube and sonicated (Sonic Dismembrator, Fisher Scientific) 3 times for 5 s with 15 s intervals of rest at 30% amplitude. Protein concentration was measured with the bicinchoninic acid (BCA) assay, and samples were frozen in aliquots at − 80 °C. Protein integrity was checked by one-dimensional SDS-PAGE. For subsequent immunoblots or ELISAs, an equal weight of brain powder was homogenized with 6 × volume per weight in cytoplasmic extraction buffer (CEB), membrane extraction buffer (MEB) from a Subcellular Fractionation kit (Pierce), or with 10 × volume per weight in RIPA buffer (150 mM NaCl, 0.1% SDS, 1% Triton-X 100, 0.5% sodium deoxycholate, 50 mM Tris, pH 8.0), supplemented with 1 × HALT protease and phosphatase inhibitor cocktail (UK286007, Thermo scientific) as previously described [47]. Brain powder suspensions were sonicated (Sonic Dismembrator, Fisher Scientific) 5 times for 2 s with 8 s intervals of rest at 30% amplitude. Brain lysates were obtained by centrifugation at 500 × rcf for 10 min at 4 °C. Protein supernatants were transferred to a new 1.5 mL Eppendorf tube. Protein concentration was measured with the bicinchoninic acid (BCA) assay and saved samples were saved at − 80 °C.

### Tandem mass tag (TMT) peptide labeling and electrostatic repulsion-hydrophilic interaction chromatography (ERLIC) fractionation

Proteolytic digestion of protein samples and cleanup was performed as previously described [14]. Briefly, protein samples were reduced with 1 mM dithiothreitol (DTT) for 30 min, alkylated with 5 mM iodoacetamide (IAA) in the dark for an additional 30 min and then diluted 8-fold with 50 mM triethylammonium bicarbonate (TEAB). Overnight digestion was performed with 1:100 (w/w) Lysyl endopeptidase (Wako) followed by an additional 12-h digestion with Trypsin at 1:50 (w/w). Peptide solutions were acidified and desalted with a C18 Sep-Pak column (Waters). A 2 μg equivalent of each sample elution was pooled and used to create a global internal standard (GIS) and all samples were dried under vacuum. Tandem mass tag (TMT) peptide labeling was performed according to manufacturer’s instructions and as previously described [14]. One batch of 10-plex TMT kits (Thermo Fisher) was used to label 8 samples and two GIS mixtures per batch. Electrostatic repulsion-hydrophilic interaction chromatography (ERLIC) offline fractionation was performed as previously described [14, 15]. Briefly, dried samples were re-suspended in 100 μL of ERLIC buffer A (90% acetonitrile with 0.1% acetic acid) and separated on a PolyWAX LP column (20 cm by 3.2 mm packed with 300 Å pore, 5 μm beads (PolyLC Inc.) and elution fractions were recovered over a 45-min gradient from 0 to 50% ERLIC buffer B (30% ACN with 0.1% FA).

### LC–MS/MS and TMT data acquisition

Assuming equal distribution of peptide concentration across all ERLIC fractions, 10 μL of loading buffer (0.1% TFA) was added to each of the fractions and 2 μL was separated on a 25 cm long 75 μm internal diameter fused silica column (New Objective, Woburn, MA) packed in-house with 1.9 μm Reprosil-Pur C_18_-AQ resin. The LC–MS/MS platform consisted of a Dionex RSLCnano UPLC coupled to an Orbitrap Fusion mass spectrometer with a Flex nano-electrospray ion source (Thermo Fisher). Sample elution was performed over a gradient of 3 to 30% Buffer B (0.1% formic acid in ACN) over 105 min (flow rate started at 300 nL/min and ended at 350 nL/min), from 30 to 60% B over 20 min at 350 nL/min, and from 60 to 99% B over 5 min at 350 nL/min. The column was equilibrated with 1% B for 10 min at a flow rate that increased from 350 nL/min to 400 nL/min. The MS was operated in positive ion mode and utilized the synchronous precursor selection (SPS)-MS3 method for reporter ion quantitation as described [90]. The full scan range was 380–1500 m/z at a nominal resolution of 120,000 at 200 m/z and automatic gain control (AGC) set to 2 × 10^5^. Collision-induced dissociation (CID)-Tandem MS/MS at 35% normalized collision energy (CE) and higher energy collision dissociation (HCD) SPS-MS3 at 65% normalized collision energy (CE) were collected at top speed with 3 s cycles. For SPS, the top 10 product ions were notched and fragmented.

### Protein identification and quantification

Raw data files from the Orbitrap Fusion were processed using Proteome Discover (version 2.1). Collected MS/MS spectra were searched against the UniProt mouse proteome database (54,489 total sequences). SEQUEST parameters were specified as: trypsin enzyme, two missed cleavages allowed, minimum peptide length of 6, TMT tags on lysine residues and peptide N-termini (+ 229.162932 Da) and carbamidomethylation of cysteine residues (+ 57.02146 Da) as fixed modifications, oxidation of methionine residues (+ 15.99492 Da) and deamidation of asparagine and glutamine (+ 0.984016 Da) as a variable modification, precursor mass tolerance of 20 ppm, and a fragment mass tolerance of 0.6 Da. Peptide spectral match (PSM) error rates were determined using the target-decoy strategy coupled to Percolator [16] modeling of true and false matches. Reporter ions were quantified from MS3 scans using an integration tolerance of 20 ppm with the most confident centroid setting. An MS2 spectral assignment false discovery rate (FDR) of less than 1% was achieved by applying the target-decoy strategy. Following spectral assignment, peptides were assembled into proteins and were further filtered based on the combined probabilities of their constituent peptides to a final FDR of 1%. In cases of redundancy, shared peptides were assigned to the protein sequence with the most matching peptides, thus adhering to principles of parsimony. In total, 8695 proteins were identified by tandem mass spectrometry. The search results and TMT quantification as well as raw LC–MS/MS files are included in the ProteomeXchange online repository with identifier (to be uploaded and assigned). Prior to data analysis, preliminary network connectivity outliers were determined as samples with connectivity beyond 3 standard deviations from the mean using Oldham's “SampleNetworks” v1.06 R script as previously published, but no cases were identified for removal [84, 102].

### Data preparation

Each TMT batch consisted of a single age group, either 3 month or 19 month, of *Grn*^+*/*+^ and *Grn*^−/−^ mice which led to some proteins being measured in one batch and not in the other. As a result, batch wise missing values for these proteins across the two TMT multiplexes led to exactly 50% missing values. To limit the effects of batch wise differences on data analysis methods, initial analysis was conducted on the 6566 unique proteins which were measured across all batches. Proteins with 50% of quantified values were later mapped into the existing network and volcano plots (2129 proteins).

### Differential expression analysis and MetaScape

Differentially enriched or depleted proteins (p ≤ 0.05) were identified by one-way ANOVA with post-hoc Tukey HSD test comparing four groups: 3-month-old *Grn*^+/+^, 3-month-old *Grn*^−/−^, 19-month-old *Grn*^+/+^ mice and 19-month-old *Grn*^−/−^ mice. Differential expression of proteins were visualized with volcano plots generated using the ggplot2 package in Microsoft R Open v3.4.2. Significantly differentially expressed proteins were determined by both having a p ≤ 0.05 and a fold change difference of greater than log_2_(1.25) or less than − log_2_(1.25) (a minimum 25% fold change).

Proteins that were significantly differentially expressed in *Grn*^+/+^ and *Grn*^−/−^ mouse brain proteomes were analyzed using MetaScape as described [136]. Briefly, differentially expressed genes were analyzed using the MetaScape web portal (https://metascape.org/) to identify enriched ontology clusters in the data set. Statistically enriched terms (i.e. GO/KEGG terms), accumulative hypergeometric p-values, and enrichment factors were calculated and used for filtering. The significant terms were hierarchically clustered into a tree based on Kappa-statistical similarities among their gene memberships, then 0.3 kappa score applied as threshold to cast the tree into term clusters.

### Weighed co-expression network analysis

Following previously described procedures of WGCNA [102], a weighted protein co-expression network was generated using the protein abundance network of 6566 unique proteins. WGCNA::blockwiseModules() function was used with the following settings: soft threshold power beta = 29, deepSplit = 4, minimum module size of 25, TOMdenom = “mean”, merge cut height of 0.07, pamStage = TRUE and a reassignment threshold of p < 0.05. Hierarchical protein correlation clustering analysis was conducted using 1‐TOM, and initial module identifications were established using dynamic tree cutting as implemented in the WGCNA::blockwiseModules() function [66]. Module eigenproteins were defined as the first principal component of coexpression module protein log_2_(abundances) [74]. The module membership measure is defined as k_ME_. K_ME_ is the pearson correlation between the expression pattern of the protein and the module eigenprotein. Bicor correlation was used for pairwise complete correlation of non-missing measurements with the cognate samples’ levels in module eigenproteins. The top correlated eigenprotein was used to assign the proteins with 50% missing values to a module, albeit with diminished confidence due to N = 8 instead of 16.

### Gene ontology (GO) and cell-type enrichment analysis

To characterize groups of differentially expressed proteins and co‐expressed proteins, we used GO Elite v1.2.5 as previously published [102] with pruned output visualized using an in‐house R script. Overrepresentation of ontologies in each module was determined by Z-score value. Enrichment of cell type across co‐expression modules was investigated by intersecting module proteins with lists of proteins known to be expressed by each cell marker [103] and assessing significance of overlap using a one‐tailed Fisher exact hypergeometric overlap test. After assessing significance, the *p*‐values were corrected by the Benjamini–Hochberg method. Cell type-specific gene lists are provided in Additional file 1: table S1.

### Mouse plasma samples

Mouse blood samples were collected by cheek vein puncture into EDTA tubes and chilled on ice for 1 h. Plasma samples were separated by centrifugation at 500 rcf for 10 min at 4 °C and stored at − 80 °C for ELISA.

### Human samples processing

#### Human brain samples

Human brain tissue was provided by the Emory Brain Tissue Bank (Emory University Goizueta Alzheimer’s Disease Research Center, Atlanta, Georgia, USA) and the Mayo Clinic Brain Bank (Jacksonville, Florida). A summary of the neuropathological and clinical descriptors of human post-mortem samples used for immunostaining and ELISA is provided as a supplementary table (Additional File 2: table S2). This included a total of frozen frontal cortex samples from FTD-*GRN* patients (n = 21, 11 males and 10 females, mean age 66.81 ± 1.66 years), cognitively normal controls (n = 23, 13 males and 10 females, mean age 62.17 ± 2.51 years), among which 5 corresponding paraffin-embedded brain sections in each group were collected. Human brain tissue was processed as described for mouse brains described above. Briefly, an equal weight of brain powder was homogenized with 10 × volume per weight in RIPA buffer and saved for protein analysis (BCA), immunoblot, and ELISA.

#### Human CSF samples

Human cerebrospinal fluid (CSF) samples were obtained from the Advancing Research and Treatment for Frontotemporal Lobar Degeneration (ARTFL) and the Longitudinal Evaluation of Familial Frontotemporal Dementia Subjects (LEFFTDS) studies (ARTFL-LEFFTDS), which were housed at the National Centralized Repository for Alzheimer Disease and Related Dementias (NCRAD). CSF was obtained by lumbar puncture from individuals with genetic mutations associated with FTD (FTD-GRN n = 13, FTD-C9orf72 n = 13, or FTD-MAPT n = 12) or cognitively normal controls (n = 14) and stored at − 80 °C before analysis. NCRAD receives government support under a cooperative agreement grant (U24 AG21886) awarded by the National Institute on Aging (NIA).

### Immunostaining and imaging

Immunohistochemistry bright field staining was performed on 5 μm human and mouse brain paraffin sections as previously described [77, 99, 122]. In brief, sections were deparaffinized in xylene and rehydrated using a descending series of ethanol concentrations (100%, 95%, 70%). To retrieve the antigens, sections were boiled in 10 mM sodium citrate buffer (pH = 6.0) using a microwave oven and cooled down for 1 h to reach room temperature. Brain tissue was treated with 3% H_2_O_2_ in deionized water for 20 min to block endogenous peroxidase activities. Slides blocked in 5% normal animal serum in 0.01 M TBS for 1 h at room temperature. Next, sections were incubated overnight at 4 °C with anti-mouse Cathepsin D (Goat, AF1029, 1:500, R&D Systems), anti-mouse Cathepsin Z (Goat, PA5-47048,1:500, Thermo Fisher Scientific), anti-mouse GPNMB (Goat, AF2330, 1:1000, R&D Systems), anti-mouse Galectin-3 (Goat, AF1197,1:1000, R&D Systems), anti-mouse progranulin (Sheep, AF2557, 1:500, R&D System), or anti-human GPNMB (Rabbit, AF2550, 1:500, R&D System) antibody. ImmPRESS HRP polymer detection kits horse anti-goat IgG (MP7405-15, Vector laboratories) or horse anti-rabbit IgG (MP-7401-15, Vector laboratories) was used for detecting primary antibodies and amplifying signals. For mouse progranulin staining, biotinylated donkey anti-sheep secondary antibody (713-065-003, 1:200, Jackson ImmunoResearch) was used. To visualize staining, diaminobenzidine (DAB) with nickel enhancement (SK4100, Vector Laboratories) was performed and hematoxylin (72511, Thermo Fisher Scientific) was used for nuclear counterstain. Finally, sections were dehydrated with increasing concentrations of ethanol (70%, 95%, 100%) and xylene for 2 min each then mounted with permanent mounting media (8312-4, Thermo Fisher Scientific). Whole slide images were captured on an Aperio AT2 Slide Scanner (Leica) and analyzed in Aperio ImageScope software.

Immunofluorescence staining was also performed on 5 μm mouse and human brain paraffin sections. Deparaffinization, rehydration and antigen retrieval steps were the same as immunohistochemistry staining above. Permeabilization was performed by incubating slides with 0.01 M TBS buffer containing 0.4%Triton-X 100 solution and 1% normal animal serum for 10 min. Non-specific binding was blocked with a one-hour incubation at room temperature with 5% normal animal serum in 0.1%Triton-X 100 in 0.01 M TBS. Autofluorescence from lipofuscin and blood vessels was blocked using Trueblack (#23007, Biotium) diluted in 70% ethanol for 30 s, followed by a rinse with TBS. Then, sections were incubated overnight at 4 °C with primary antibody: anti-mouse GPNMB (Goat, AF2330, 1:1000, R&D Systems), anti-mouse Galectin-3 (Goat, AF1197, 1:1000, R&D Systems), anti-Iba1 (Rabbit, mAb17198, 1:1000, Cell Signaling Technology), anti-GFAP (Rabbit, Z0334, 1:1000, Agilent Dako) or anti-NeuN (Rabbit, mAb24307, 1:1000, Cell Signaling Technology) antibody. The following day, sections were incubated with fluorescent secondary antibodies: Donkey anti-rabbit Alexa Fluor 488 (R37118, 1:500, Invitrogen), and Donkey anti-rabbit Alexa Fluor 647 (A-21447, 1:500, Invitrogen) for 1 h at room temperature. DAPI (#62248, 1:1000, Thermo Fisher Scientific,) was used to stain the nucleus and Prolong Gold anti-fade mountant (P36930, Thermo Fisher Scientific) was used for cover slipping to protect fluorescent signals.

### Immunoblot

Immunoblots were performed as previously described [27, 55, 72]. Briefly, human and mouse brain running samples were prepared in loading buffer with 50 mM 2-Mercaptoethanol (BME) followed by denaturing at 70 °C for 15 min. Protein samples were separated on Bio-Rad 4–20% 18-well midi gels at 100 V and transferred to a 0.2 µm nitrocellulose membrane using the Bio-Rad Trans-blot Turbo system. Membranes were stained with total protein stain (926-11010, Licor) and imaged on the Odyssey Fc (Licor) to access transfer efficiency and normalize protein abundance between samples. After blocking with Licor Odyssey blocking buffer for 1 h at room temperature, membranes were incubated overnight at 4 °C with primary antibody: anti-mouse Cathepsin D (Goat, AF965, 1:500, R&D Systems), anti-mouse Cathepsin Z (Goat, PA5-47048, 1:1000, Thermo Fisher Scientific), anti-mouse GPNMB (Goat, AF2330, 1:1000, R&D Systems), anti-mouse Galectin-3 (Goat, AF1197, 1:1000, R&D Systems), anti-human Galectin-3 (Goat, AF1154, 1:1000, R&D Systems), or anti-human GPNMB (Goat, AF2550, 1:1000, R&D Systems) antibody. All primary antibodies were diluted in 1:1 TBST/Blocking buffer. Alpha-tubulin (Rabbit, 1878-1, 1:10,000, Epitomics) and beta-actin (Rabbit, ab8227, 1:1000, Abcam) were used as loading controls. Near-infrared fluorescent secondary antibodies (diluted in TBST) or HRP-conjugated (diluted in 0.5% milk in TBST) antibodies were incubated for 1 h at room temperature: Donkey anti-goat Alexa Fluor 680 (A21084, 1:10,000, Invitrogen), goat anti-rabbit 790 (A11369, 1:10,000, Invitrogen), donkey anti-rabbit 680 (A21109, 1:10,000, Invitrogen), or donkey anti-goat HRP (705-035-003, 1:15,000, Jackson ImmunoResearch) were used. For HRP visualization, blots were incubated in WesternSure PREMIUM Chemiluminescent Substrate (926-95010, Licor) for 5 min before imaging. Near-infrared or chemiluminescent blots were imaged using the Odyssey Fc (Licor) and analyzed by Image Studio software 5.2.

### ELISA

General ELISA protocols were performed as previously described [55, 63, 64]. Levels of GPNMB and galectin-3 in tissue were quantified using ELISAs Duosets according to the manufacturer’s protocol: human GPNMB (AF2550, R&D Systems), mouse GPNMB (AF2330, R&D Systems), human Galectin-3 (DY1154, R&D Systems), or mouse Galectin-3 (DY1197, R&D Systems). Additional ELISA reagents were from ELISA reagents kit 2 (DY008, R&D Systems). Briefly, ELISA plates were coated with 100 µL/well coating antibody diluted in coating buffer overnight at room temperature. After washing with 1 × wash buffer 3 times, plates were blocked with 1X reagent diluent 300 µL/well at least 1 h. Human brain RIPA lysates, CSF samples, mouse brain RIPA lysates or mouse plasma were diluted at 1:20, 1:4, 1:50, or 1:10 respectively, in 1X reagent diluent and added to plates with standard at 100 µL/well and incubated for 2 h. Next, 100 µl of diluted detection antibody was added per well. 100 µL of the working dilution of Streptavidin-HRP was added to each well and plates were incubated for 20 min in the dark. Then, 100 µL of substrate solution was added to each well and incubated for 15 min while still avoiding light. To stop the reaction, 50 µL of 2 N sulfuric acid was added to each well. Finally, ELISA plate sample absorbance (450 nm signal and 570 nm for background correction reading) was measured on an Epoch plate reader (BioTek) and processed using Gen5 software (BioTek). All samples were run in duplicate and values fell within the standard curve generated with recombinant human or mouse GPNMB or galectin-3 protein provided in DuoSet ELISA kits. Protein measurements in human and mouse brain samples were normalized to the amount of total protein added per well.

### Statistical analysis

Standard curve generation and statistical analyses were performed by GraphPad Prism 8.0. An unpaired student's t-test for two groups and one-way or two-way ANOVA for more than two groups were used to generate *p* values. All quantitative data are presented as mean ± SEM and significance levels are denoted ****p < 0.0001, ***p < 0.001, **p < 0.01 and *p < 0.05.

## Results

### Proteomic analysis of *Grn*^+/+^ and *Grn*^−/−^ mouse brain

**Fig. 1:**
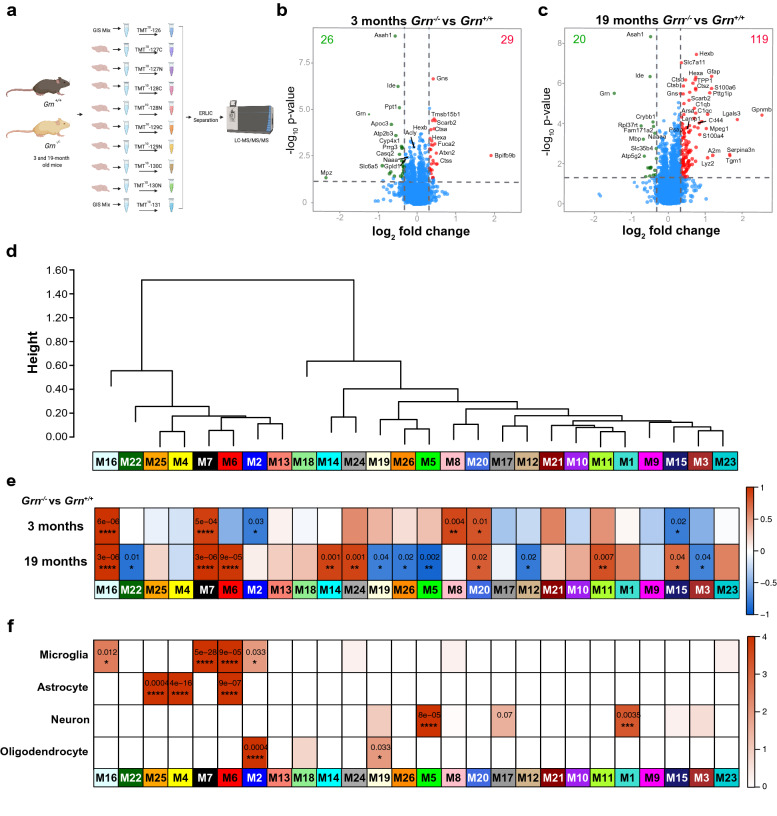
Proteomics and network analysis of *Grn*^+/+^ and *Grn*^−/−^ mouse brain. **a** Schematic overview of experimental design. Quantitative proteomic analysis of the cerebral cortex of 3-month-old (n = 4 *Grn*^+/+^, n = 4 *Grn*^−/−^) and 19-month-old (n = 4 *Grn*^+/+^, n = 4 *Grn*^−/−^) mice was performed using Tandem Mass Tag (TMT) isobaric labeling and synchronous precursor selection-based MS3 (SPS-MS3) mass spectrometry. **b**, **c** Volcano plots illustrating differentially expressed proteins in 3- and 19-month-old mouse brains. Relative protein abundance (log2 *Grn*^−/−^/ *Grn*^+/+^) plotted against significance level (− log10 P-value), showing significantly (*p* < 0.05) decreased (Grn^−/−^/ *Grn*^+/+^ ratio < log_2_(− 1.25); green) and increased (*Grn*^−/−^/ *Grn*^+/+^ ratio > log_2_(1.25); red) proteins in *Grn*^−/−^ mice. **d** WGCNA cluster dendrogram generated by hierarchical clustering of highly co-expressed genes followed by identifying 26 distinct modules coded by different colors. **e** Two-color heatmap is showing the relationship between modules and the bicor correlation of genotype. Significance levels are ****p < 0.0001, ***p < 0.001, **p < 0.01 and *p < 0.05. **f** Significance of cell type overlap is shown by one-color heatmap with P values. Relevance between each module and cell type was assessed by protein module overlap with known mouse microglia, astrocyte, neuron and oligodendrocyte markers. Significance levels are ****p < 0.0001, ***p < 0.001, **p < 0.01 and *p < 0.05

We used unbiased proteomics to quantify how PGRN deficiency changes the mouse brain proteome with age to provide insight into the function of PGRN and how PGRN deficiency causes neurodegeneration. This was accomplished using Tandem Mass Tag (TMT) isobaric labeling and synchronous precursor selection-based MS3 (SPS-MS3) mass spectrometry to perform quantitative proteomic analysis of the cerebral cortex in 3- and 19-month old *Grn*^+/+^ and *Grn*^−/−^ mice (Fig. 1a). The use of 10-plex TMT labeling followed by off-line electrostatic repulsion-hydrophilic interaction chromatography (ERLIC) fractionation prior to LC–MS/MS enabled identification of 8,695 unique proteins. Next, we determined which proteins were differentially enriched or depleted (p ≤ 0.05; one-way ANOVA-Tukey HSD) in the brain proteomes of 3- or 19-month old *Grn*^+*/*+^ compared to age-matched *Grn*^−/−^ mice (Fig. 1b, c); Additional file 1: table S3). In the 3-month *Grn*^−/−^ mouse brain samples, 29 proteins increased and 26 proteins decreased in abundance compared to *Grn*^+*/*+^ mice of the same age (Fig. 1b). Gene ontology (GO) analysis of all significantly altered proteins *Grn*^−/−^ mouse brain using MetaScape revealed a significant enrichment (− log10(p) > 10) of proteins involved in lysosome function (Kegg pathway: mmu04142) and glycosphingolipid metabolism (Reactome: R-MMU-1660662) (i.e. Gns, Scarb2, Hexa, Hexb, Fuca 2, Pppt1, and Ctsa) [136]. GO analysis focusing on downregulated proteins in the 3-month *Grn*^−/−^ brain proteome identified a significant enrichment (− log10(p) > 4) of proteins involved in lipid catabolism (Acly, Apoc3, Asah1, Gpld1, Ppt1, Naaa; GO:0016042), which may indicate that PGRN deficiency causes impairment of the lysosomal degradation and recycling of lipids.

In 19-month-old *Grn*^−/−^ mice, 119 proteins were increased and 20 proteins were decreased compared to *Grn*^+*/*+^ mice. In the brains of 19-month-old *Grn*^−/−^ mice we found that an even larger number of lysosomal proteins were increased, including glycoprotein NMB (GPNMB), which is the most upregulated protein in aged *Grn*^−/−^ mice (Fig. 1c). In addition to lysosome alterations, GO analysis revealed novel upregulated pathways in 19 month old *Grn*^−/−^ mice including proteins involved in gliogenesis (C1qa, Dbi, Gfap, P2rx4, Stat3, Bin1, Zfp365) as well as inflammation, complement, and coagulation cascades (C1qa, C1qb, C1qc, C4b, Itgb2, and A2m) [136]. Thus, our initial analysis of the mouse brain proteome reveals an early dysregulation of proteins involved in lysosomal function and lipid metabolism in 3-month-old *Grn*^−/−^ mice, which is exacerbated with age, leading to upregulation of proteins involved in the innate immune response and inflammation.

### Co-expression protein analysis with *Grn*^+/+^ and *Grn*^−/−^ mouse

Next, we performed weighted co-expression network analysis (WGCNA) on the *Grn*^+*/*+^ and *Grn*^−/−^ mouse brain proteome datasets to determine the relationship between the changes in protein abundance we observed and biological pathways and cell types [66]. Network analysis identified 26 modules of strongly co-expressed groups of proteins (Fig. 1d; Additional file 1: table S4, table S5). Each protein co-expression module is defined by its first principal component, an eigenprotein, which is also the most representative weighted protein expression pattern across samples for a group of co-expressed proteins [102]. Modules were clustered based on relatedness defined by the correlation distance between eigenproteins (Fig. 1d). We calculated the relationship between modules and the biweight midcorrelation (bicor) [106] of eigenproteins to genotype and age of *Grn* mice and identified a number of modules that significantly correlated (Fig. 1e). Comparison of modules in the *Grn*^+*/*+^ 19-month versus *Grn*^+*/*+^ 3-month data set or *Grn*^−/−^ 19-month versus 3-month *Grn*^−/−^ data set identified many of the same modules, suggesting this analysis identifies common or shared protein modules that are generally altered during aging (Additional file 3: Fig. S1, online resource).

**Fig. 2:**
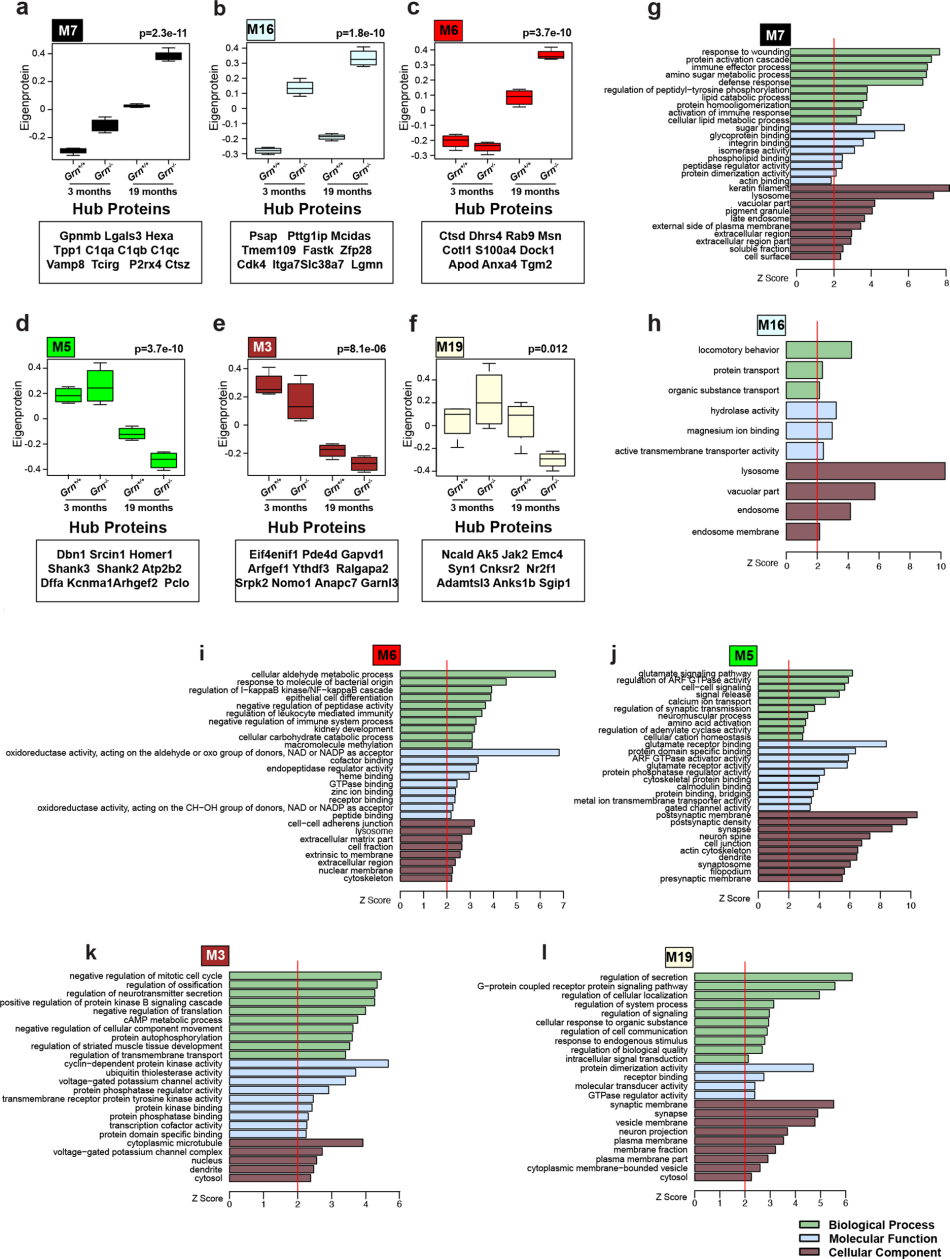
Modules that correlate to *Grn* deficiency and age highlight dysregulation of proteins involved in the lysosome, inflammation, and neuronal synaptic function. **a**–**c** Box plots illustrate eigenprotein values for modules (M7, M16, and M6 red) that are upregulated in 3- or 19-month-old *Grn*^−/−^ mice compared to *Grn*^+*/*+^ mice. **d**–**f** Box plots of M5, M3 and M19 modules that have decreased eigenprotein values in *Grn*^−/−^ versus *Grn*^+*/*+^ in 19-month-old mice. Values were analyzed by two-way ANOVA. Gene ontology (GO) enrichment analysis, calculated using GO Elite v1.2.5, of proteins in modules, highlights proteins involved in: **g** immune effector process, glycoprotein-binding, lysosome (M7), **h** protein transport, hydrolase activity, lysosome (M16),** i** oxidoreductase activity, regulation of NF kappa B cascade, lysosome (M6), **j** glutamate signaling, postsynaptic density, neuron spice and dendrite (M5), **k** regulation of neurotransmitter secretion, voltage-gated potassium channel activity, cytoplasmic microtubule (M3), **i** regulation of creation, synaptic membrane and synapses (M19). Light-green bars: biological process, light-blue bars: molecular function, brown-bars: cellular component

In contrast, comparison of 3-month old *Grn*^−/−^ mice to 3-month old *Grn*^+*/*+^ mice identified 6 modules that significantly correlated to *Grn* deficiency (Fig. 1e). The ontology of these modules are described by a structural component, function, or biological process: modules M2 myelin, M7 lysosome/immune response, M8-synapse, M15-cation channel/transport, M16 lysosome/hydrolase activity, M20 ribosome (Fig. 2 and Additional file 1: table S5; Additional file 3: Fig. S2–5, online resource). The M2 myelin and M15-cation channel module were modestly, but significantly (*p* = 0.003 and *p* = 0.02, respectively), decreased in the 3-month cohort. The M16 (Fig. 2b, h**)** and M7 (Fig. 2a, g) lysosome modules were the most upregulated modules and the most significantly correlated (*p* = 6 × 10^−6^ and *p* = 5 × 10^−4^, respectively) to *Grn* deficiency in the 3-month old *Grn*^−/−^ brain proteome, indicating these modules are key drivers of disease pathogenesis.

Comparison of 19-month old *Grn*^−/−^ mice to 19-month old *Grn*^+*/*+^ mice identified 14 modules with significant correlation to *Grn* deficiency (Fig. 1e). Three of the same modules (M7, M16, and M20) that were increased in 3-month old *Grn*^−/−^ mice were similarly increased in the 19-month old group. Novel modules that were altered in the 19-month old *Grn*^−/−^ brain proteome include: modules M3-microtubule/cell-cycle (Fig. 2e, k), M5-postsynaptic/glutamate signaling (Fig. 2d, j), M6-lysosome/oxidation–reduction (Fig. 2c, i), M11-ribosome/translation, M12-nucleus/nucleic acid metabolism, M14-GPCR signaling, M19-synaptic membrane/secretion (Fig. 2f, i), M22-pyruvate/acetyl–CoA metabolism, M24-axon/defense response, and M26-membrane/mitochondria (Fig. 2a–l and Additional file 3: Fig. S2–5, online resource). The three most significantly upregulated modules M6 (Fig. 2c, i; *p* = 9 × 10^−5^), M7 *(*Fig. 2a, g; *p* = 3 × 10^−6^), and M16 (Fig. 2b, h; *p* = 3 × 10^−6^) are related to lysosome function, further implicating the importance of this pathway to normal PGRN function and alteration following *Grn* deficiency. Interestingly, the most significantly decreased modules in 19-month old *Grn*^−/−^ brain contain proteins important for postsynaptic function and glutamate signaling (M5, Fig. 2d, j; *p* = 0.002), synaptic membrane/secretion (M19, Fig. 2f, i*; p* = 0.04), and mitochondria function (M22, *p* = 0.01; M26, *p* = 0.02; Additional file 3: Fig. S5).

Next, we asked if the changes we observed in specific modules were driven by changes in cell types. To do this, we evaluated whether a given module was enriched in marker proteins for particular CNS cell types using a previously established cell-type specific proteome derived from the mouse brain [53](Fig. 1f; Additional file 1: table S1). We observed significant enrichment of neuronal proteins in the M5 postsynaptic module (Fig. 1f; Dbn1, Shank2, Shank1, Camk4) and M1 module (Fig. 1f; Dpysl5, Madd, Sugp2, Lamb1). Further, oligodendrocyte protein markers were enriched in the M2 myelin module (Fig. 1f; Plp1, Mbp, Mog, cldn11) and the M19 synaptic membrane module (Fig. 1f; Ncald, Anks1b, Jak2, Gpr158). The observation that neuronal (M5, *p* = 0.002) and oligodendrocyte (M19, *p* = 0.033) modules are decreased specifically in the brain of 19-month, not 3-month, *Grn*^−/−^ mouse brain suggests that neuronal loss and demyelination are a late-stage consequence of *Grn* deficiency.

We also found significant enrichment of microglial proteins in the M16 lysosome module (Fig. 1f; Lgmn, Uap1l1, Stk11ip), the M7 lysosome module (Fig. 1f; Gpnmb, P2rx4, Lgals3, Tcirg, Hexb), the M6 lysosome module (Fig. 1f; Cotl1, Msn, Ctsd, Tgm2), and the M2 myelin module (Fig. 1f; Aldh3b1). Protein markers of astrocytes were significantly enriched in the M6 module (Fig. 1f; Gja1, Dhrs4, Acsf2, Nadk2) as well as the M4 (Fig. 1f; Gstt1, Ccbl2, Abat, Aldh4a1, Gcsh) and M25 modules (Fig. 1f; Fam213a, Vcl, Hrsp12, Acadvl, Hadha), both of which contain mitochondrial proteins. Our observation that M7 and M16 modules are both enriched in microglia markers and lysosomal proteins, and are the most significantly elevated modules in the 3-month old *Grn*^−/−^ brain proteome, suggests that dysregulation of microglial lysosomes may be the earliest pathologic change in *Grn*^−/−^.

### *Aged Grn*^−/−^ mouse brains accumulate lysosomal proteins and markers of neuroinflammation

**Fig. 3:**
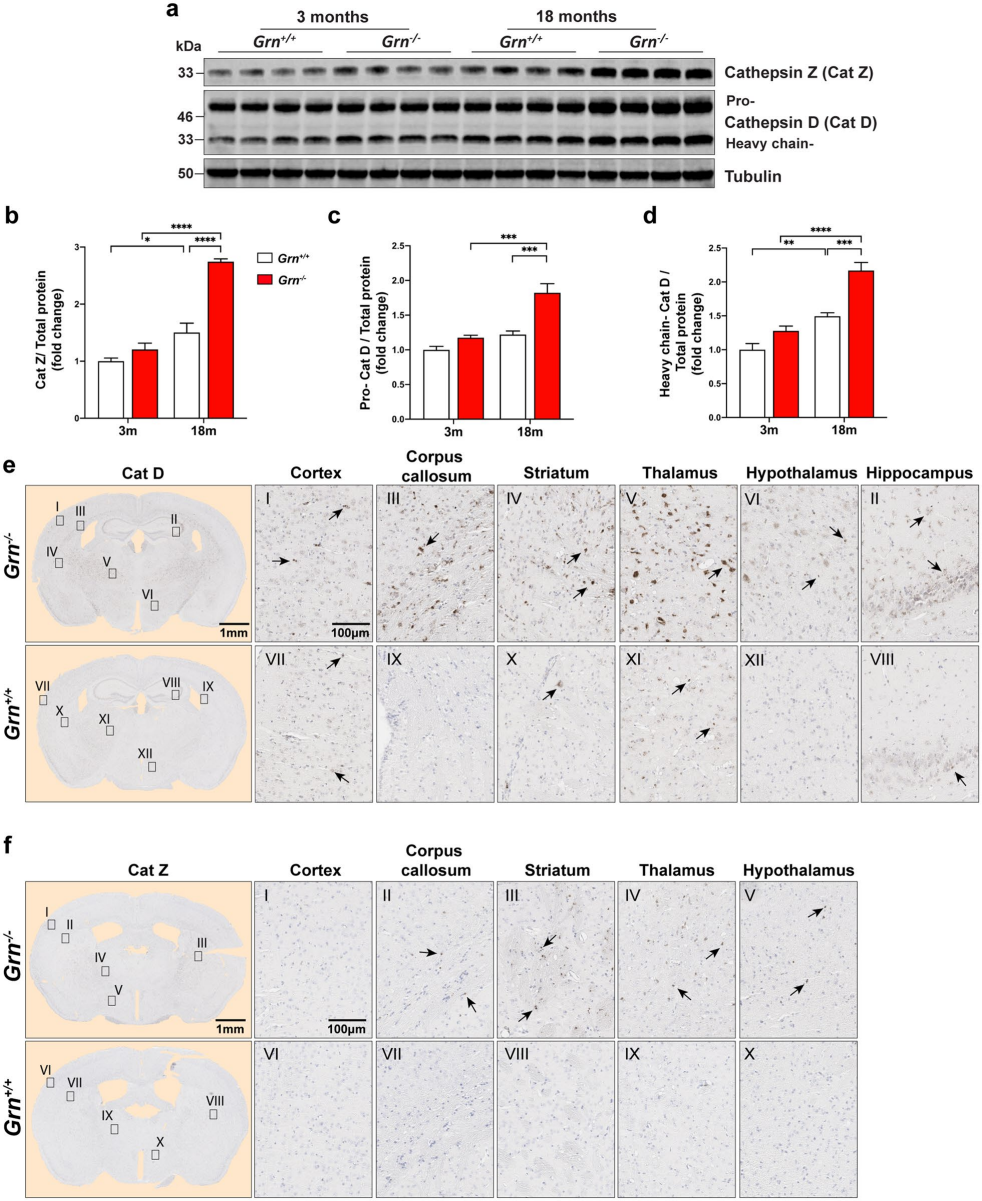
The lysosomal proteins, cathepsin D (Cat D) and Cathepsin Z (Cat Z), are elevated in *Grn*^−/−^ mouse brains. **a** Images of immunoblots of lysates of *Grn*^+*/*+^ and *Grn*^−/−^ mouse brains at 3- (n = 4) and 18- (n = 4) months of age probed for Cat (33 kDa), pro Cat D (~ 48 kDa) and the mature heavy chain of Cat D (~ 33 kDa). Alpha-tubulin used as a loading control. **b**–**d** Fold change of signal intensity determined by comparing the 3-month-old *Grn*^+/+^ group mean signal intensity measurement value of Cat Z and Cat D bands from (**a**) and normalized to total protein signal of blot. **f** Immunohistochemistry staining of Cat D in 19-month-old *Grn*^+*/*+^ and *Grn*^−/−^ mouse brains. Region specific Cat D expression in the cortex (I, VII), hippocampus (II, VIII), corpus callosum (III, IX), striatum (IV, X), thalamus (V, XI) and hypothalamus (VI, XII). **g** Region specific Cat Z expression in the cortex (I, VI), corpus callosum (II, VII), striatum (III, VIII), thalamus (VI, IX), and hypothalamus (V, X) of 19-month-old *Grn*^+*/*+^ and *Grn*^−/−^ mouse brains. Scale bars (1 nm to 100 µm) are labeled in images and quantitative data shown as mean ± SEM, *p* < 0.05; **p < 0.01; ***p < 0.001; ****p < 0.0001

Because GO and network analysis revealed that dysregulation of the lysosomal pathway was an early and highly significant event in the *Grn*^−/−^ mouse brain proteome, we focused on validating and examining these changes using biochemical and immunological orthogonal approaches. First, we examined the expression levels and neuroanatomical location of two lysosomal proteins, cathepsin Z (protein abbreviation, Cat Z; gene, *Ctsz*) and cathepsin D (protein abbreviation, Cat D; gene *Ctsd*), enriched in modules M7 and M6, respectively. We performed immunoblotting of whole brain lysates from 3-month (n = 8) and 18-month-old (n = 8) *Grn*^+*/*+^ and *Grn*^−/−^ mice. First, we examined Cat Z, a unique cysteine cathepsin, previously implicated in neurodegenerative diseases, but not examined in the context of FTD or PGRN biology [3, 13, 110]. Levels of Cat Z increased in *Grn*^+*/*+^ (1.5-fold) and *Grn*^−/−^ (2.3-fold) in whole brain lysates of 18-month mouse brain as measured by quantitative immunoblotting (Fig. 3a, b). Next, we examined the levels of Cat D, a key lysosomal aspartyl protease that has been suggested to play an important role in PGRN function [9, 115]. We observed a significant increase in both the pro- (~ 46 kDa) and heavy chain (~ 33 kDa) isoforms of Cat D (Fig. 3a, c, d). There were no significant differences in Cat Z and Cat D levels between *Grn*^+*/*+^ and *Grn*^−/−^ mouse brain at 3 months, suggesting PGRN deficiency leads to an age-dependent increase of both Cat Z and Cat D. Next, we performed immunostaining on 19-month-old (n = 14) *Grn*^−/−^ brain coronal sections to determine the regions of Cat Z and Cat D expression and upregulation. We found strong immunostaining of Cat D in the thalamus, corpus callosum, striatum, and hippocampus in *Grn*^−/−^ mouse brains (Fig. 3e). Immunostaining of Cat Z was also increased in the thalamus, corpus callosum, and striatum of *Grn*^−/−^ mice, but to a lesser extent than Cat D (Fig. 3f). Taken together, immunoblotting and immunostaining of *Grn*^−/−^ mouse brains confirm our proteomic results and demonstrate that PGRN deficiency leads to an age-dependent increase in the lysosomal proteins Cat Z and Cat D.

### An age-dependent increase of GPNMB and Galectin-3 in Granulin Knockout mice

**Fig. 4:**
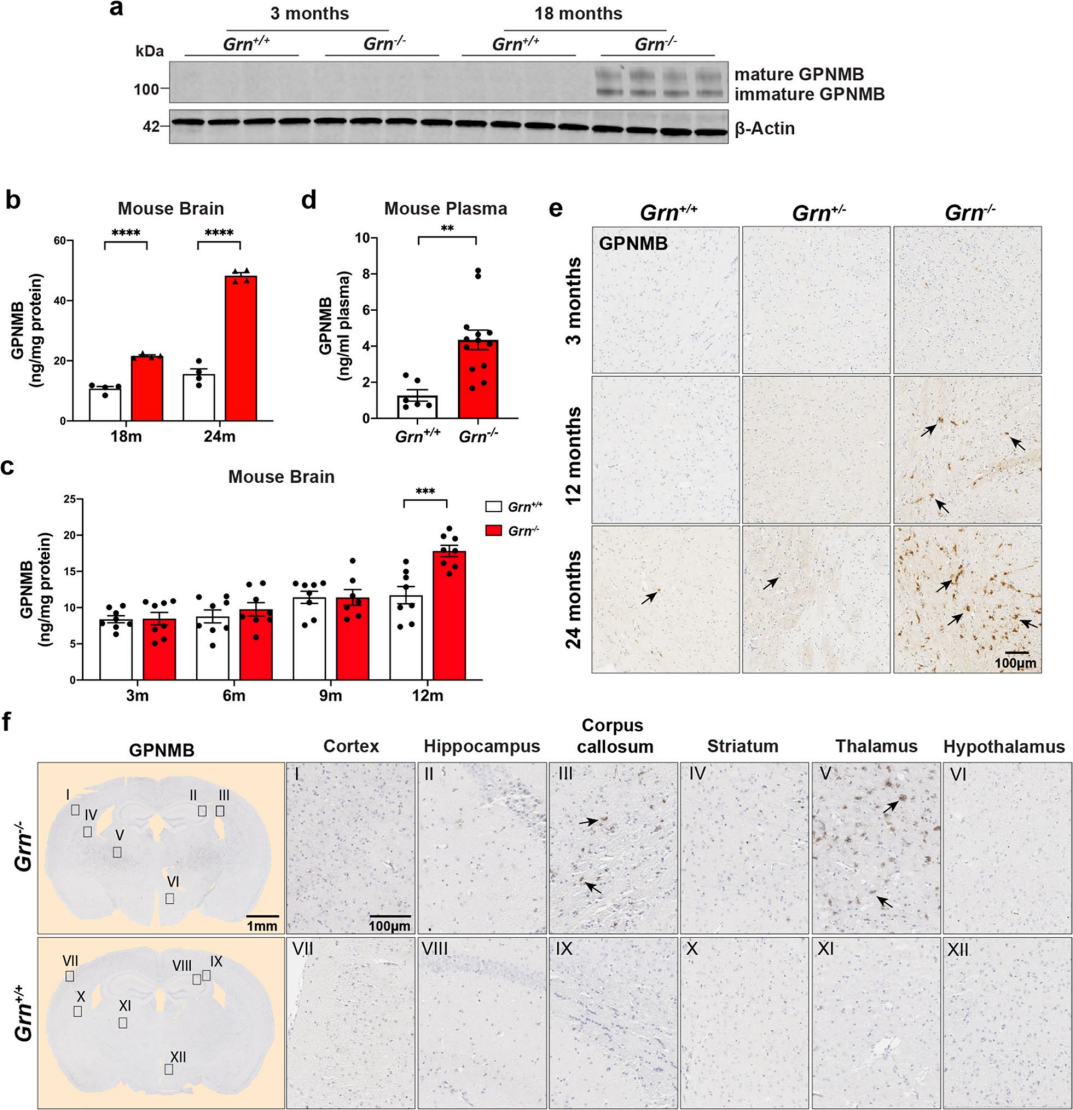
GPNMB levels increase in the brain and plasma of *Grn*^−/−^ mice with age. **a** Representative immunoblots of immature and mature (glycosylated) GPNMB in mouse brain lysates of *Grn*^+/+^ and *Grn*^−/−^ mice at 3- and 18-months of age. Beta-actin used as loading control. **b** Levels of GPNMB (ng/mg protein) in mouse brain lysates measured by ELISA. Tissue from *Grn*^+/+^ (white) and *Grn*^−/−^ (red) mice at 18-months (n = 4 per group) and 24-months (n = 4 per group), respectively. Data analyzed by two-way ANOVA. **c** ELISA quantification of mouse brain GPNMB levels (ng/mg of brain protein) in *Grn*^+*/*+^ (white) and *Grn*^−/−^ (red) mice at 3-, 6-, 9- and 12-month ages (n = 8 per group). Values analyzed by two-way ANOVA. **d** Levels of GPNMB in 19-month old *Grn*^+/+^ (white) and *Grn*^−/−^ (red) mouse plasma measured by ELISA. Data (n = 6 for *Grn*^+/+^ and 13 for *Grn*^−/−^) analyzed using an unpaired student t-test. **e** Representative sections of *Grn*^+/+^, *Grn*^+/−^, and *Grn*^−/−^ mouse thalamus at 3-, 12-, and 24-months of age immunostained for GPNMB. **f** 19-month-old *Grn*^+/+^ and age-matched *Grn*^−/−^ mouse brain coronal sections were stained with anti-GPNMB antibody. GPNMB staining shown for multiple brain regions (cortex (I, VII), hippocampus (II, VIII), corpus callosum (III, IX), striatum (IV, X), thalamus (V, XI) and hypothalamus (VI, XII)). Scale bars (1 nm to 100 µm) are labeled in images and quantitative data shown as mean ± SEM, *p* < 0.05; **p < 0.01; ***p < 0.001; ****p < 0.0001

**Fig. 5:**
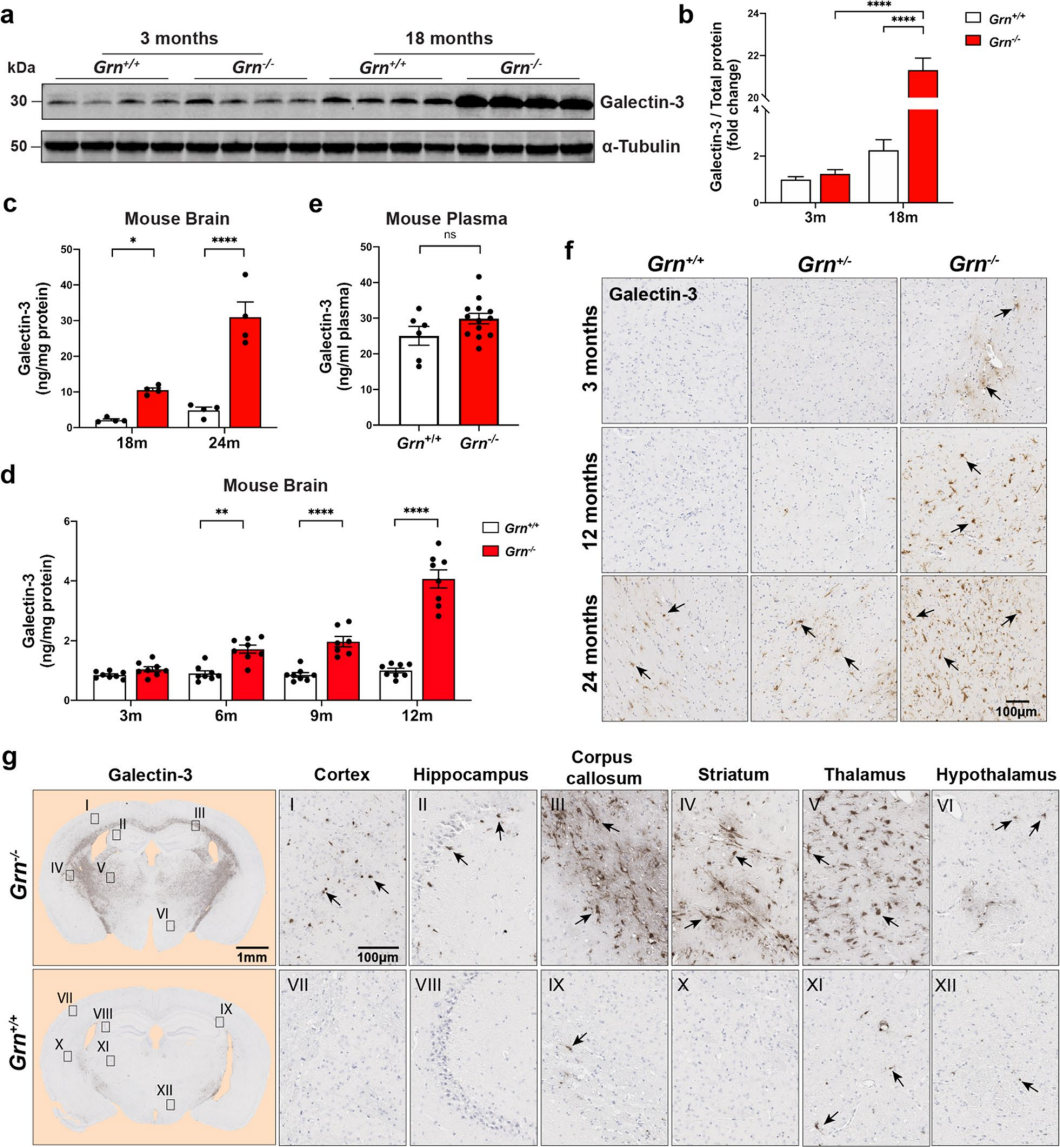
Galectin-3 levels are robustly increased with age in *Grn*^−/−^ mouse brain**. a**, **b** Representative immunoblot (left) and quantification (right) of galectin-3 levels in the brain lysates of 3- and 18-month-old *Grn*^+/+^ mice and age-matched *Grn*^−/−^ mice (n = 4 mice each group). Band values were normalized by total protein values and analyzed by two-way ANOVA. **c** Levels of mouse brain galectin-3 (ng/mg of protein) in lysates were quantified by ELISA. Galectin-3 levels were normalized to total protein added to assay and results analyzed by two-way ANOVA. **d** Galectin-3 protein measured in 19-month-old *Grn*^+/+^ and *Grn*^−/−^ mice plasma samples by ELISA. Data analyzed using unpaired t-test. **e** Brain sections from *Grn*^+/+^, *Grn*^+/−^, *Grn*^−/−^ mice at 3-, 12- and 24-months of age were immunostained with galectin-3 antibody. Representative images of thalamus from each genotype are shown. **f** 19-month-old *Grn*^+/+^ and age-matched *Grn*^−/−^ mice brain coronal sections were stained with anti-GPNMB antibody and images from multiple regions (cortex (I, VII), hippocampus (II, VIII), corpus callosum (III, IX), striatum (IV, X), thalamus (V, XI) and hypothalamus (VI, XII)) are shown. Scale bars (1 nm to 100 µm) are labeled in images and quantitative data shown as mean ± SEM, *p* < 0.05; **p < 0.01; ***p < 0.001; ****p < 0.0001

Next, we focused on examining the expression of transmembrane glycoprotein NMB (GPNMB) and galectin-3 (*LGALS3*), because these were two of the most enriched proteins in the *Grn*^−/−^ brain proteome, and are key hub proteins of module M7 (Figs. 1d, e, 2a). Additionally, excess levels of GPNMB and galectin-3 have not been previously associated with neurodegeneration associated with FTD, suggesting they might be involved in pathogenesis or serve as biomarkers. Towards this goal, we examined GPNMB and galectin-3 expression levels in *Grn*^+*/*+^ and *Grn*^−/−^ at different ages using immunoblotting and immunohistochemistry (Figs. 4, 5).

First, we were unable to detect mouse GPNMB in either 3-month old *Grn*^+*/*+^ or *Grn*^−/−^ mouse brains by immunoblot analysis of detergent brain extracts. However, in 18-month-old *Grn*^−/−^ mouse brain lysates, we detected strong immunoreactive bands for mature (glycosylated; top band) and immature GPNMB compared to *Grn*^+*/*+^ mice (Fig. 4a). This result confirms our proteomic data that GPNMB is a highly upregulated protein in aged 18-month-old *Grn*^−/−^ mouse brain. Next, we used ELISAs to quantify the level of GPNMB in whole brain lysates from 18- (n = 8) and 24-month-old (n = 8) *Grn*^+*/*+^ and *Grn*^−/−^ mice. GPNMB levels were significantly increased in both 18-month-old *Grn*^−/−^ (2.0-fold; *p* < 0.0001) and 24-month-old *Grn*^−/−^ (3.1-fold; *p* < 0.0001) brain tissue compared to age-matched *Grn*^+*/*+^ brain tissue (Fig. 4b). Subsequently, we asked when GPNMB is first elevated in *Grn*^−/−^ mouse tissue. To do this, we quantified GPNMB levels in *Grn*^+*/*+^ and *Grn*^−/−^ mouse brain tissue at 3- (n = 16), 6- (n = 16), 9- (n = 16), and 12- (n = 16) months of age by ELISA (Fig. 4c). Based on this analysis, GPNMB levels are first significantly increased at 12-months of age in *Grn*^−/−^ mouse brains. Then, we asked if an increase in GPNMB levels could be detected in the blood. Indeed, GPNMB levels were increased ~ 2-fold (*p* < 0.01) in 19-month-old *Grn*^−/−^ mice plasma compared to *Grn*^+*/*+^ (Fig. 4d).

Next, we performed immunohistochemical staining for GPNMB on sagittal brain sections from 3-, 12-, and 24-month-old *Grn*^+*/*+^, *Grn*^+/−^, and *Grn*^−/−^ mice to determine where GPNMB expression is most upregulated in the brain (Fig. 4e). We noted elevated GPNMB staining in the thalamus of *Grn*^−/−^ mice at 12 and 24 months of age compared to both *Grn*^+*/*+^ and *Grn*^+/−^ brains, in agreement with our ELISA results. Further, there was no appreciable difference in GPNMB staining between *Grn*^+*/*+^ and *Grn*^+/−^ mice at any age. Finally, we completed a more extensive survey of GPNMB staining on coronal brain sections of 19-month old *Grn*^+*/*+^ and *Grn*^−/−^ mice. We observed strong increases in GPNMB immunoreactivity in the corpus callosum and the thalamus and lower levels of increase in the hippocampus, cortex, and striatum (Fig. 4f).

Next, we repeated the same analyses for galectin-3. Based on quantitative immunoblotting, galectin-3 levels were 21-fold higher (*p* < 0.0001) in 18-month-old *Grn*^−/−^ mouse brain lysate compared to age-matched *Grn*^+*/*+^ samples (Fig. 5a, b). In order to quantify galectin-3 protein levels more accurately, RIPA lysates of 18- and 24-month old mouse brain were analyzed using ELISAs. Galectin-3 levels were significantly increased in *Grn*^−/−^ mouse brains in both ages compared to age-matched *Grn*^+*/*+^ mice (Fig. 5a, b). At 18-months, galectin-3 levels were 2.12 ± 0.64 ng/mg protein in *Grn*^+*/*+^ mice and 10.53 ± 1.25 ng/mg protein in *Grn*^−/−^ mice. At 24-months, galectin-3 levels increased to 4.85 ± 1.78 ng/mg protein in *Grn*^+*/*+^ mice and 30.99 ± 8.55 ng/mg protein in *Grn*^−/−^ mice (Fig. 5a, b). Subsequently, we asked what age is galectin-3 first elevated in *Grn*^−/−^ mouse tissue. We quantified galectin-3 levels in *Grn*^+*/*+^ and *Grn*^−/−^ mouse brain tissue at 3- (n = 16), 6- (n = 16), 9- (n = 16), and 12- (n = 16) months of age by ELISA (Fig. 5d). Based on this analysis, galectin-3 levels are first significantly elevated at 6 months of age in *Grn*^−/−^ mouse brains (*p* < 0.01) and continue to increase with age (Fig. 5d). Interestingly, unlike GPNMB, we did not detect a significant change in galectin-3 levels in *Grn*^−/−^ plasma compared to *Grn*^+*/*+^ plasma (Fig. 5e).

Next, we asked where galectin-3 expression is most upregulated in the brain. First, we performed immunohistochemical staining for galectin-3 on sagittal brain sections from 3-, 12-, and 24-month-old *Grn*^+*/*+^, *Grn*^+/−^, and *Grn*^−/−^ mice. We noted elevated galectin-3 staining in the thalamus of *Grn*^−/−^ mice as early as 3 months of age compared to both *Grn*^+*/*+^ and *Grn*^+/−^ brains (Fig. 5f). This is an earlier time point than our ELISA results, which used whole brain lysates, which may mask subtle changes in focal regions of the brain. Further, there was no appreciable difference in galectin-3 staining between *Grn*^+*/*+^ and *Grn*^+*/*^ mice at any age. Finally, we completed a more extensive survey of galectin-3 staining on coronal brain sections of 19-month old *Grn*^+*/*+^ and *Grn*^−/−^ mice (Fig. 5g). We observed very strong galectin-3 immunoreactivity throughout the myelin tracts of the corpus callosum, the striatum, and the thalamus. Galectin-3 immunoreactivity was also increased in the cortex, hippocampus, and hypothalamus, although the staining was less widespread and more punctate. Overall, these results confirm our initial proteomics data that GPNMB and galectin-3 levels are specifically increased in the brains of *Grn*^−/−^ mice.

### Cellular localization of GPNMB and galectin-3 in *Grn*^−/−^ mice

**Fig. 6:**
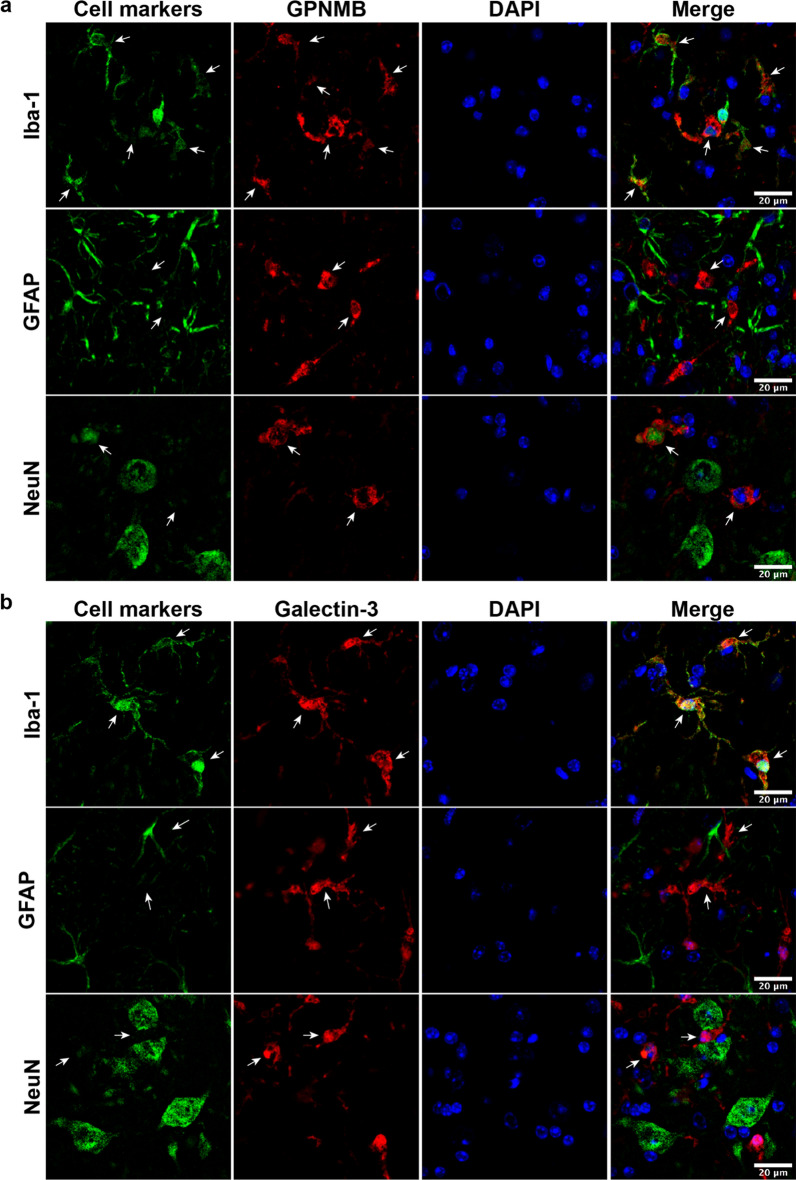
GPNMB and galectin-3 co-localize with Iba-1 positive microglia cells in *Grn*^−/−^ mouse brain. **a** Representative immunofluorescent co-staining for different cell markers (Iba-1, microglia; GFAP, astrocytes; NeuN, neurons) are shown in green, GPNMB (red), and nuclei (DAPI; blue) in brains of 19-month-old *Grn*^−/−^ mice. **b** Representative immunofluorescent co-staining for different cell markers (Iba-1, microglia; GFAP, astrocytes; NeuN, neurons) are shown in green, galectin-3 (red), and nuclei (DAPI; blue) in brains of 19-month-old *Grn*^−/−^ mice. Scale bars (20 µm) labeled in images

Next, we aimed to determine which cells express GPNMB and galectin-3 in 19-month-old *Grn*^−/−^ mice brains. We performed double immunofluorescent staining for GPNMB or galectin-3 with antibody markers for microglia (Iba-1), astrocytes (GFAP), and neurons (NeuN) (Fig. 6a, b). We detected strong co-localization between GPNMB, galectin-3, and Iba-1, indicating microglia express both proteins in aged *Grn*^−/−^ mouse brain. This further supports our network-based proteomic analysis that detected a strong overlap between module M7, which contains GPNMB, galectin-3, and microglia cell markers (Fig. 1f). In contrast, we did not detect co-localization between GFAP-positive astrocytes and GPNMB or galectin-3. Similarly, we did not detect robust co-localization of GPNMB or galectin-3 signal in NeuN-positive neurons. On occasion, we observed GPNMB co-staining in some cells that were weakly positive for NeuN. In summary, double immunofluorescent suggests that microglia are the major source of GPNMB and galectin-3 expression in *Grn*^−/−^ mouse brains.

### GPNMB and Galectin-3 are increased in FTD-*GRN* brain tissue

**Fig. 7:**
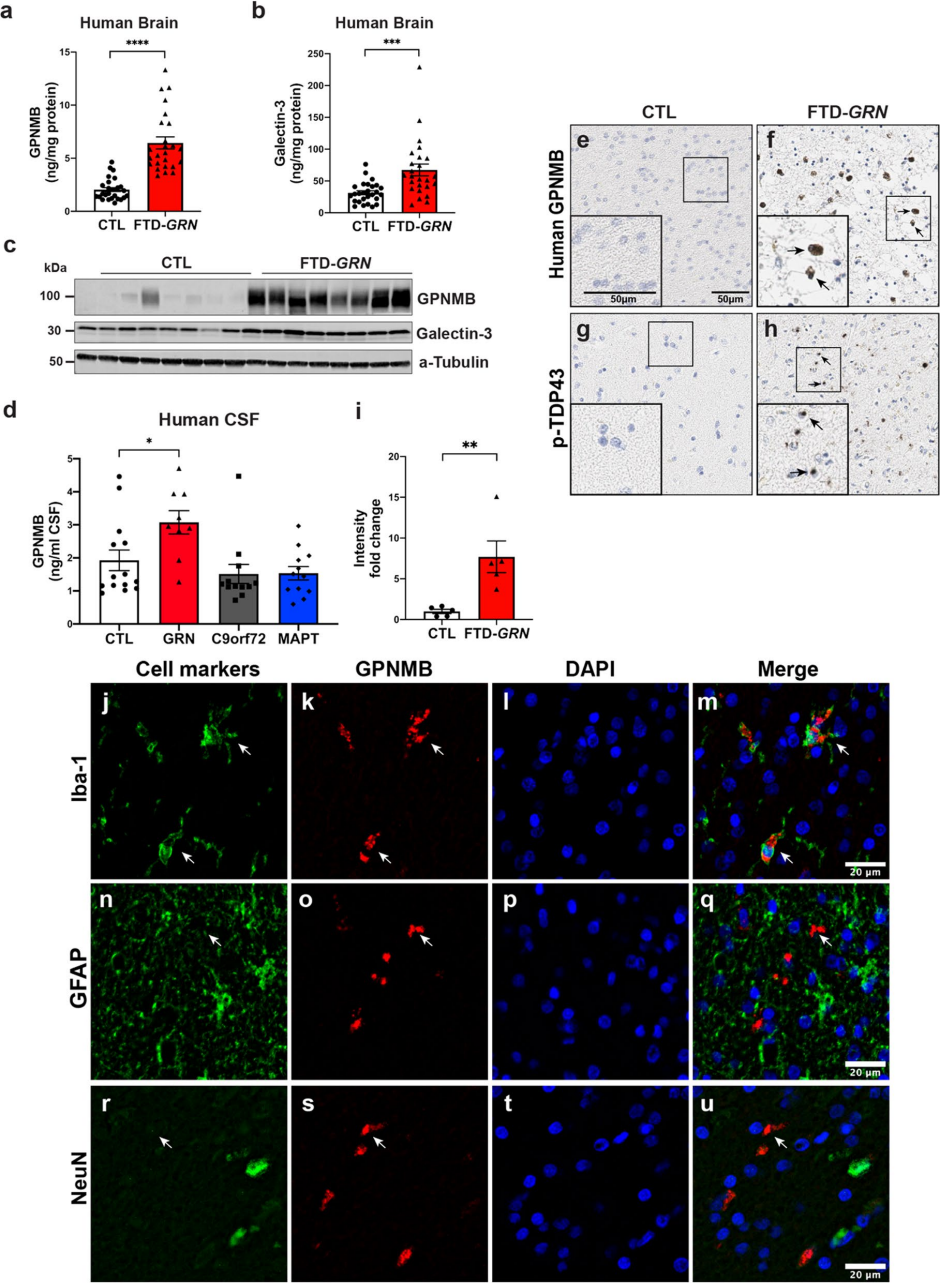
GPNMB and galectin-3 levels are elevated in FTD-*GRN* brains. **a**, **b** GPNMB and galectin-3 levels (ng/mg protein) were measured in frontal lobe tissue lysates generated from cognitively normal controls (CTL; n = 27) and FTD-*GRN* patients (n = 25). Data analyzed using unpaired t-test. **c** Representative immunoblots for GPNMB and galectin-3 in frontal lobe lysates from cognitively normal controls (n = 8) and FTD-*GRN* (n = 8) patients. **d** GPNMB levels (ng/mL) in CSF samples form cognitively normal controls (n = 14), FTD-*GRN* (n = 9), FTD-*C9orf72* (n = 12) and FTD-*MAPT* (n = 12) samples quantified by ELISA. Data analyzed using one-way ANOVA. **e**, **f** GPNMB immunostaining was performed on frontal lobe tissue sections from cognitively normal controls (n = 5) (**e**) and FTD-GRN (n = 5) (**f**) patients. **g**, **h** Immunostaining for p-TDP 43 was stained on adjacent sections from identical samples in **e**, **f** as marker of FTLD pathology. **i** GPNMB staining intensity in human brain sections (**e**, **f**) were measured and presented as fold change. Representative immunofluorescence staining for cell markers (green) (**j**, **n**, **r**), GPNMB (red) (**k**, **o**, **s**), DAPI (blue) (**i**, **p**, **t**) in paraffin sections of brains from FTD-*GRN* cases. Iba-1, GFAP, NeuN used for markers of human microglia, astrocytes, and neurons respectively. GPNMB and Iba-1 signals overlap (arrow) (**m**) whereas, no overlapping signal was observed in co-staining with GFAP or NeuN (**q**, **u**). Scale bars were labeled in the images. Data analyzed by unpaired t-test. Scale bars (20 µm) labeled in images and quantitative data are shown as mean ± SEM, *p < 0.05; **p < 0.01; ***p < 0.001; ****p < 0.0001

Finally, we asked if the pathologic changes we observed in aged *Grn*^−/−^ mice also occur in the brains of FTD-*GRN* patients. We generated detergent lysates of the frontal lobe from 21 FTD-*GRN* samples and 23 cognitively normal controls. Initial immunoblotting of a subset of these cases suggested an increase in both GPNMB and galectin-3 in FTD-*GRN* brain homogenate compared to controls. Next, in order to more accurately quantify all samples, we measured human GPNMB and galectin-3 using sandwich ELISAs. Levels of GPNMB (*p* < 0.0001) and galectin-3 (*p* < *0.001*) were both significantly increased in FTD-*GRN* brain homogenates compared to controls (Fig. 7a, b).

Subsequently, we examined the distribution of GPNMB in more detail in FTD-*GRN* brains. First, we examined GPNMB expression in the frontal lobe of five FTD-*GRN* cases compared to age-matched, cognitively normal controls. Specificity of the anti-GPNMB antibody for immunohistochemistry was validated using recombinant GPNMB protein to block staining (Additional file 3: Fig. S6, online resource). Interestingly, the strongest signal for GPNMB was detected in the white matter of the frontal lobe in FTD-*GRN* brains (Fig. 7e, f). Intensity of GPNMB immunoreactivity was 6.5-fold higher (*p* < 0.01) in the frontal lobes of FTD-*GRN* brains compared to matched regions from the brains of cognitively normal controls (Fig. 7e, f, Fig. S7a–d). Adjacent sections of FTD-*GRN* brains, but not controls, were immunopositive for phosphorylated TDP-43, confirming the presence of FTLD pathology in these cases [54, 81] (Fig. 7g, h, Fig. S7e–h). Then, to address cell-type specificity, we performed double immunofluorescent staining of GPNMB and antibody markers for microglia (Iba-1), astrocytes (GFAP), and neurons (NeuN) (Fig. 7j–u). We detected strong co-localization between GPNMB and Iba-1 in human FTD-*GRN* brains. No co-localization between GPNMB and GFAP or NeuN signal was detected. These data suggest microglia are the predominant source of GPNMB expression in human FTD-*GRN* brains.

Next, we asked if GPNMB levels were increased in FTD bio-fluids. We obtained CSF from patients with pathogenic genetic FTD mutations collected from the Advancing Research and Treatment for Frontotemporal Lobar Degeneration (ARTFL) and Longitudinal Evaluation of Familial Frontotemporal Dementia Subjects (LEFFTDS) studies that were stored in the National Centralized Repository for Alzheimer’s Disease and Related Dementias (NCRAD) [95, 97]. GPNMB protein levels were quantified with sandwich ELISAs in CSF samples from individuals that were cognitively normal (controls; n = 14) and individuals with known mutations that cause FTD (FTD-*GRN* n = 13, FTD-*C9orf72* n = 13, or FTD-*MAPT* n = 12). GPNMB levels were significantly up regulated (*p* < 0.05) in the FTD-*GRN* group (3.07 ± 0.35 ng/mL CSF) compared to controls (1.92 ± 0.31 ng/mL CSF) (Fig. 7d). In contrast, there was no significant difference in GPNMB levels between controls and FTD-*C9orf72* or FTD-*MAPT* CSF samples.

## Discussion

Although PGRN haploinsufficiency is a well-established cause of FTD, the function of PGRN and the pathogenic cascade caused by PGRN deficiency that ultimately leads to neurodegeneration is still unclear [22, 86]. In this study, we performed deep proteomic analysis of whole brains from *Grn*^−/−^ mice, which recapitulate many features of FTD, including behavioral impairments and neuroinflammation [5, 10, 60, 82].

The first major finding is that the levels of lysosomal proteins are altered in young 3-month old c mouse brains. Our proteomics data is the first to find lysosomal dysregulation at such an early time point. This is likely due to the sensitivity of TMT-based proteomics and the known discrepancy between RNA transcript levels and protein expression [102]. In particular, we detected significant increases in well-established proteins that localize to the lumen or membrane of the lysosome including glucosamine (N-Acetyl)-6-Sulfatase (GNS), prosaposin (Psap), lysosomal integral membrane protein-2 (Limp-2/Scarb2), cathepsin A (CtsA), and hexosaminidases A and B (Hexa; Hexb). Importantly, the genes encoding all of these proteins harbor pathogenic mutations that cause lysosome storage disorders [91]. Furthermore, expression of these and other lysosomal genes are under control of the transcription factors TFEB and TFE3 and are up-regulated under conditions of lysosomal stress or dysfunction [93, 98, 101]. Thus, early lysosome dysfunction in *Grn*^−/−^ mice may manifest by increased expression of many lysosomal proteins. In agreement with our findings, the mRNA transcripts of many of the lysosomal genes we have identified have been reported to be increased in older *Grn*^−/−^ mice [42, 135]. Taken together, our data indicate that lysosomal dysfunction occurs early in *Grn*^−/−^ brains and likely initiates pathogenesis and eventual neurodegeneration.

Our findings also provide insight into the function of PGRN. Recently, a potential role of PGRN in lysosome homeostasis has emerged based on the discovery that multiple cases of homozygous *GRN* mutation carriers develop neuronal ceroid lipofuscinosis-11 (CNL11), a lysosomal storage disease [4, 18, 50, 105]. We, and other labs, have found that PGRN is trafficked to the lysosome and processed by cathepsins into granulins, which may be bioactive [47, 68, 133]. However, the function of PGRN and granulins within the lumen of the lysosome is still unclear. Because PGRN deficiency causes impairment of lysosomal protease activity and accumulation of lipofuscin [125], one possibility is that loss of PGRN/granulins directly or indirectly decrease the levels and/or activity of a lysosomal hydrolase.

With this idea in mind, our observation that a subset of the most significantly downregulated proteins (Acly, Apoc3, Asah1, Gpld1, Ppt1, Naaa) in the 3 month *Grn*^−/−^ brain proteome are involved in lysosomal lipid catabolic process is important. Of these proteins two, ATP-citrate lyase (Acly) and apolipoprotein (apo) C-III (Apoc3), are involved in lipogenesis and lipid homeostasis [30, 94]. Intriguingly, the remaining proteins are all involved in lipid degradation pathways. N-acylsphingosine amidohydrolase 1 (Asah1) hydrolyzes sphingolipid ceramides into sphingosine and free fatty acids in the lysosome [87]. N-Acylethanolamine Acid Amidase (Naaa) degrades bioactive fatty acid amides, such as N-palmitoylethanolamine [114]. Palmitoyl-protein thioesterase 1 (Ppt1) removes thioester-linked fatty acyl groups like palmitate from modified cysteine residues during lysosomal degradation of proteins [59]. Finally, glycosylphosphatidylinositol specific phospholipase D1 (Gpld1) hydrolyzes the inositol phosphate linkage in proteins anchored by phosphatidylinositol glycans (GPI-anchor) to release proteins from the membrane and has recently been linked to the cognitive benefits of exercise for the aged brain [49]. Of note, earlier work found that loss of PGRN leads to an accumulation of polyunsaturated triacylglycerides and reduced diacylglycerides and phosphatidylserines in *Grn*^−/−^ fibroblasts and mouse brains [29]. Our data provide additional evidence that PGRN deficiency leads to impaired degradation and recycling of lipids in the lysosome. Further work is necessary to determine if PGRN or granulins have a direct or indirect role in lysosomal lipid metabolism and homeostasis.

Our analysis of differentially expressed proteins in 19-month-old *Grn*^−/−^ brains uncovered an even greater number of lysosomal proteins that are increased including Hexa, Hexb, Tpp1, and Fuca2. Intriguingly, proteins involved in inflammation and immune response (i.e. complement genes C1qa, C1qb, and C1qc) are significantly increased. Our data agrees with earlier work demonstrating that multiple *Grn*^−/−^ mouse models have age-dependent microgliosis and astrogliosis throughout the brain including the cortex, hippocampus, and thalamus [1, 37, 82, 127, 132]. The upregulation of C1qa, C1qb, and C1qc we observe is consistent with previous transcriptomic data from *Grn*^−/−^ mice, which reported upregulation in C1qa, C1qb, and C1qc before the onset of neurodegenerative features [29, 70]. Further, microglia isolated from 5.5-month old *Grn*^−/−^ mice are activated and upregulate expression of genes associated with a microglial neurodegenerative phenotype (MGnD) [41]. Thus, gliosis and a robust inflammatory response is a consistent observation in multiple *Grn*^−/−^ mouse models that increases with age, suggesting this is may be a downstream consequence initially triggered by lysosome dysfunction.

We also identified novel proteins that increase with age in the brains of *Grn*^−/−^ mice. In particular, two of the most strongly up-regulated proteins were GPNMB (also known as osteoactivin) and galectin-3, suggesting they may play an important role in disease progression. Our co-localization studies demonstrate that GPNMB and galectin-3 are strongly expressed by microglia in aged *Grn*^−/−^ mouse brain. GPNMB is a widely expressed transmembrane type I protein that has been implicated in many cellular functions including cell adhesion, cell migration, cell proliferation, and cell differentiation [113]. Intriguingly, variants in *GPNMB* are associated with Parkinson’s disease, highlighting the potential importance of GPNMB broadly in neurodegenerative diseases [52, 89]. Galectin-3 is a member of the lectin family, contains a carbohydrate-recognition-binding domain that mediates binding of β-galactosides, and plays an important role modulating inflammation [28]. Galectin-3 has also been implicated in brain innate immunity associated with neurodegeneration [16]. High levels of both GPNMB and galectin-3 levels have been found in the brain of 5xFAD mice [51] as well also the brains of PD and AD patients [11, 61, 75, 100]. Additionally, levels of GPNMB were elevated in grey and white matter of spinal cord of ALS patients [79].

We observed the strongest increases in GPNMB and galectin-3 expression in the thalamus of *Grn*^−/−^ mouse, providing additional evidence that this region of the brain is particular vulnerable to loss of PGRN [70]. We also found high levels of GPNMB and galectin-3 in the corpus callosum, which is highly myelinated. Abundant galectin-3 staining was found along white matter tracts in the cortex and striatum, suggesting that the expression and upregulation of GPNMB and galectin-3 in microglia is related to myelin and white matter.

Importantly, myelin basic protein (MBP), a major component of white matter, is decreased in *Grn*^−/−^ mouse brain. Furthermore, modules enriched for oligodendrocyte proteins (M19, *p* = 0.033) and neuronal and synaptic proteins (M5, *p* = 0.002) are not significantly decreased until 19-months of age in *Grn*^−/−^ mouse brain, indicating that synaptic and neuronal loss and demyelination are a late-stage consequence of *Grn* deficiency. Our proteomics data is supported by a previous report that found defective myelination in the cerebral cortex of *Grn*^−/−^ mice [108]. Our discovery that GPNMB and galectin-3 are elevated in the white matter of frontal lobe of FTD-*GRN* cases provides additional clinical relevance suggesting alterations of these proteins may contribute to neurodegeneration.

Intriguingly, demyelination that is observed as white matter hyperintensities (WMH) on MRI brain scans is a frequent and specific occurrence in FTD cases caused by *GRN* mutations [20, 107, 128]. Our data provide additional support to the idea that FTD-*GRN* cases are especially vulnerable to demyelination and loss of white matter, although the exact mechanism is unclear. One possibility is that aberrant microglial activation due to PGRN deficiency, caused by lysosome dysfunction and protein aggregation, leads to phagocytosis, synaptic pruning, and de-myelination that eventually manifests as white matter damage and WMH. Intriguingly, microglia expressing either GPNMB or galectin-3 have been implicated in the process of myelin phagocytosis and demyelination [46, 69, 109]. Taken together, we find that increases in microglial GPNMB and galectin-3 levels are correlated with demyelination. Currently it is unclear if GPNMB and galectin-3 upregulation is a cause or consequence of this process.

Lack of specific and sensitive biomarkers are another roadblock in the development of therapies for FTD [40]. Importantly, appropriate biomarkers are useful for monitoring disease progression as well as assessing the efficacy of potential drugs. Elevated levels of neurofilament light chain (Nfl) in CSF and plasma are one promising biomarker for symptomatic FTD patients that harbor mutations in *GRN*, *C9orf72*, or *MAPT* [118]. However, Nfl is not specific to FTD-*GRN* and is increased in several other neurodegenerative diseases [124]. In this study, we report our finding of elevated levels of GPNMB in aged *Grn*^−/−^ mouse plasma as well as CSF from human FTD patients with *GRN* mutations. Previous reports found that GPNMB was elevated in the serum of type I Gaucher disease [138], but not significantly different in AD patients [51], compared to healthy controls. Because GPNMB levels were only increased in CSF from *GRN* mutation carriers, but not *C9* or *MAPT* cases, GPNMB levels could serve as a biomarker to differentiate between FTD caused by *GRN* mutantions or other FTD genes.

It is currently unlcear why GPNMB is elevated in the microglia of *Grn*^−/−^ mice and human FTD-*GRN* patients. One intriguing possibility is that upregulation of GPNMB expression is driven by lysosome dysfunction. This idea is supported by previous reports that lysosomal stress, induced by chemical inhibition of lysosome acidification or function, causes upregulation of GPNMB in macrophages [34, 111]. GPNMB is also elevated in the substantia nigra of patients with Parkinson's disease [75], a neurodegenerative disease increasingly linked to lysosome dysfcuntion [56, 83, 123]. Moreover, chemical [75] or genetic inhibition [61] of β-glucocerebrosidase (GBA; GCase) activity leading to the accumulation of glucosylceramides also causes increased expression of GPNMB. Progranulin deficiency reduces glucocerebrosidase activity [116, 134] suggesting that the accumulation of glucosylceramide or other sphingolipids could be a proximal cause of GPNMB upregulation in the *Grn*^−/−^ mouse brain, an idea that needs further investigation. Although the precise mechanism that causes GPNMB upregulation in progranulin deficiency is unclear, our data suggest that measurement of GPNMB levels in the CSF could be used to monitor changes in microglial activation and response to therapies in FTD-*GRN* patients, similar to substrate reduction therapy in lysosome storage disorders [71, 78, 119]. One limitation of our data is a small sample size and lack of longitundal testing. Thus, further studies are necessary to investigate the utility, specificity, and sensitivity of GPNMB as a biomaker in FTD and related neurodegenerative diseases. Moreover, it will be important to determine if GPNMB and galectin-3 expression in microglia is deleterious, which would open a new therapeutic target for FTD and other diseases with PGRN deficiency.

## Conclusion

Our results demonstrate the utility of a systems biology approach in understanding a complex disease like FTD. We were able to relate changes in the *Grn*^−/−^ mouse brain proteome to known phenotypic signatures of FTD. Further analysis of these proteomic changes across age also provided insight on the mechanism(s) in which neurodegeneration occurs as result of PGRN deficiency. We identified novel proteins in the *Grn*^−/−^ mouse proteome that are decreased at 3-months, which suggest an impairment of lysosomal metabolism of lipids, including sphingolipids, which are particularly important for neuronal survival [2, 14]. Lysosomal dysregulation is exacerbated with age in the *Grn*^−/−^ mouse brain leading to neuroinflammation, synaptic loss, and decreased markers of oligodendrocytes, myelin, and neurons. For the first time, we identified increased levels of two proteins, galectin-3 and GPNMB, which have not been linked to FTD or PGRN deficiency previously and may serve as novel biomarkers or drug targets. Previous data demonstrate that upregulation of GPNMB and galectin-3 in microglia can be beneficial or harmful depending on the context, timing, and disease [12, 15, 16, 62, 109]. Further studies are necessary to understand the contribution of GPNMB and galectin-3 to FTD and related neurodegenerative disease. In summary, our findings support the idea that insufficiency of PGRN and granulins in humans cause FTD through lysosomal dysfunction and neuroinflammation and suggest novel therapeutic approaches.

## Supplementary information

**Additional file 1.** Compilation of multiple supplementary tables. Table S1 is a list of cell type-specific protein markers used to identify enrichment of cell types in WGCNA modules. Table S3 is a summary of differential protein expression in *Grn*^−/−^ (KO) compared to *Grn*^+/+^ (WT) mouse brain. Table s4 is a compilation of proteins identified in mouse brain proteome and their correlation to WGCNA modules. Table S5 is a summary of the enrichment of Gene Ontology (GO) terms for each module in WGCNA network.

**Additional file 2.** Table S2 summarizing the neuropathological, clinical diagnosis, age, sex, and other characterizes of human post-mortem samples used for immunostaining and ELISA.

**Additional file 3.** Compilation of multiple supplementary figures (S1–S7). Fig. S1 is a two-color heatmap showing the relationship between WGCNA modules and the bicor correlation of age. Fig. S2 contains multiple Box plots of WGCNA modules (M1, M2, M4, M8, M9, M10, M11, M12, M13, M14, M15, M17, M18, M20, M21, M22, M23, M24, M25, and M26). Fig. S3 displays Igraphs of modules (M3, M5, M6, M7, M16, and M19) that are associated with knockout (*Grn*^−/−^) status and corresponding gene symbols as nodes. Fig. S4 displays Igraphs of modules (M1, M2, M4, M8, M9, M10, M11, M12, M13, M14, M15, M17, M18, M20, M21, M22, M23, M24, M25, and M26 modules) associated with knockout (*Grn*^−/−^) status and corresponding gene symbols as nodes. Fig. S5 summarizes the gene ontology (GO) enrichment analysis of proteins in M1, M2, M4, M8, M9, M10, M11, M12, M13, M14, M15, M17, M18, M20, M21, M22, M23, M24, M25, and M26 modules. Light-green bars: biological process, light-blue bars: molecular function, brown-bars: cellular component. Fig. S6 is an immunoadsorption validation experiment demonstrating an anti-human GPNMB antibody binds antigen specifically on human brain sections. Fig. S7 GPNMB immunostaining was performed on frontal lobe tissue sections from four FTD-GRN patients.

## Data Availability

Analysis of the proteomic data generated during this study are included in this published article and electronic supplementary files. The raw proteomic datasets used and analyzed during the current study are available from the corresponding author on reasonable request and will be uploaded to a central publicly available data repository.

## References

[1] Ahmed Z, Sheng H, Xu YF, Lin WL, Innes AE, Gass J, Yu X, Wuertzer CA, Hou H, Chiba S (2010). Accelerated lipofuscinosis and ubiquitination in granulin knockout mice suggest a role for progranulin in successful aging. Am J Pathol.

[2] Alessenko AV, Albi E (2020). Exploring sphingolipid implications in neurodegeneration. Front Neurol.

[3] Allan ERO, Campden RI, Ewanchuk BW, Tailor P, Balce DR, McKenna NT, Greene CJ, Warren AL, Reinheckel T, Yates RM (2017). A role for cathepsin Z in neuroinflammation provides mechanistic support for an epigenetic risk factor in multiple sclerosis. J Neuroinflamm.

[4] Almeida MR, Macario MC, Ramos L, Baldeiras I, Ribeiro MH, Santana I (2016). Portuguese family with the co-occurrence of frontotemporal lobar degeneration and neuronal ceroid lipofuscinosis phenotypes due to progranulin gene mutation. Neurobiol Aging.

[5] Arrant AE, Onyilo VC, Unger DE, Roberson ED (2018). Progranulin gene therapy improves lysosomal dysfunction and microglial pathology associated with frontotemporal dementia and neuronal ceroid lipofuscinosis. J Neurosci.

[6] Baker M, Mackenzie IR, Pickering-Brown SM, Gass J, Rademakers R, Lindholm C, Snowden J, Adamson J, Sadovnick AD, Rollinson S (2006). Mutations in progranulin cause tau-negative frontotemporal dementia linked to chromosome 17. Nature.

[7] Bang J, Spina S, Miller BL (2015). Frontotemporal dementia. Lancet.

[8] Bateman A, Cheung ST, Bennett HPJ (2018). A brief overview of progranulin in health and disease. Methods Mol Biol.

[9] Beel S, Moisse M, Damme M, De Muynck L, Robberecht W, Van Den Bosch L, Saftig P, Van Damme P (2017). Progranulin functions as a cathepsin D chaperone to stimulate axonal outgrowth in vivo. Hum Mol Genet.

[10] Boddaert J, Wils H, Kumar-Singh S (2018). Methods to investigate the molecular basis of progranulin actions on brain and behavior in vivo using knockout mice. Methods Mol Biol.

[11] Boza-Serrano A, Ruiz R, Sanchez-Varo R, Garcia-Revilla J, Yang Y, Jimenez-Ferrer I, Paulus A, Wennstrom M, Vilalta A, Allendorf D (2019). Galectin-3, a novel endogenous TREM2 ligand, detrimentally regulates inflammatory response in Alzheimer's disease. Acta Neuropathol.

[12] Boza-Serrano A, Ruiz R, Sanchez-Varo R, García-Revilla J, Yang Y, Jimenez-Ferrer I, Paulus A, Wennström M, Vilalta A, Allendorf D (2019). Galectin-3, a novel endogenous TREM2 ligand, detrimentally regulates inflammatory response in Alzheimer’s disease. Acta Neuropathol.

[13] Brandenstein L, Schweizer M, Sedlacik J, Fiehler J, Storch S (2016). Lysosomal dysfunction and impaired autophagy in a novel mouse model deficient for the lysosomal membrane protein Cln7. Hum Mol Genet.

[14] Breiden B, Sandhoff K (2019). Lysosomal glycosphingolipid storage diseases. Annu Rev Biochem.

[15] Budge KM, Neal ML, Richardson JR, Safadi FF (2018). Glycoprotein NMB: an emerging role in neurodegenerative disease. Mol Neurobiol.

[16] Butovsky O, Weiner HL (2018). Microglial signatures and their role in health and disease. Nat Rev Neurosci.

[17] Cairns NJ, Neumann M, Bigio EH, Holm IE, Troost D, Hatanpaa KJ, Foong C, White CL, Schneider JA, Kretzschmar HA (2007). TDP-43 in familial and sporadic frontotemporal lobar degeneration with ubiquitin inclusions. Am J Pathol.

[18] Canafoglia L, Morbin M, Scaioli V, Pareyson D, D'Incerti L, Fugnanesi V, Tagliavini F, Berkovic SF, Franceschetti S (2014). Recurrent generalized seizures, visual loss, and palinopsia as phenotypic features of neuronal ceroid lipofuscinosis due to progranulin gene mutation. Epilepsia.

[19] Caroppo P, Camuzat A, Guillot-Noel L, Thomas-Anterion C, Couratier P, Wong TH, Teichmann M, Golfier V, Auriacombe S, Belliard S (2016). Defining the spectrum of frontotemporal dementias associated with TARDBP mutations. Neurol Genet.

[20] Caroppo P, Le Ber I, Camuzat A, Clot F, Naccache L, Lamari F, De Septenville A, Bertrand A, Belliard S, Hannequin D (2014). Extensive white matter involvement in patients with frontotemporal lobar degeneration: think progranulin. JAMA Neurol.

[21] Cenik B, Sephton CF, Kutluk Cenik B, Herz J, Yu G (2012). Progranulin: a proteolytically processed protein at the crossroads of inflammation and neurodegeneration. J Biol Chem.

[22] Chitramuthu BP, Bennett HPJ, Bateman A (2017). Progranulin: a new avenue towards the understanding and treatment of neurodegenerative disease. Brain.

[23] Chou CC, Zhang Y, Umoh ME, Vaughan SW, Lorenzini I, Liu F, Sayegh M, Donlin-Asp PG, Chen YH, Duong DM (2018). TDP-43 pathology disrupts nuclear pore complexes and nucleocytoplasmic transport in ALS/FTD. Nat Neurosci.

[24] Cruts M, Gijselinck I, van der Zee J, Engelborghs S, Wils H, Pirici D, Rademakers R, Vandenberghe R, Dermaut B, Martin JJ (2006). Null mutations in progranulin cause ubiquitin-positive frontotemporal dementia linked to chromosome 17q21. Nature.

[25] Culouscou JM, Carlton GW, Shoyab M (1993). Biochemical analysis of the epithelin receptor. J Biol Chem.

[26] Deleon J, Miller BL (2018). Frontotemporal dementia. Handb Clin Neurol.

[27] Deng Q, Holler CJ, Taylor G, Hudson KF, Watkins W, Gearing M, Ito D, Murray ME, Dickson DW, Seyfried NT (2014). FUS is phosphorylated by DNA-PK and accumulates in the cytoplasm after DNA damage. J Neurosci.

[28] Díaz-Alvarez L, Ortega E (2017). The many roles of galectin-3, a multifaceted molecule, in innate immune responses against pathogens. Mediat Inflamm.

[29] Evers BM, Rodriguez-Navas C, Tesla RJ, Prange-Kiel J, Wasser CR, Yoo KS, McDonald J, Cenik B, Ravenscroft TA, Plattner F (2017). Lipidomic and transcriptomic basis of lysosomal dysfunction in progranulin deficiency. Cell Rep.

[30] Feng X, Zhang L, Xu S, Shen AZ (2020). ATP-citrate lyase (ACLY) in lipid metabolism and atherosclerosis: an updated review. Prog Lipid Res.

[31] Finch N, Baker M, Crook R, Swanson K, Kuntz K, Surtees R, Bisceglio G, Rovelet-Lecrux A, Boeve B, Petersen RC (2009). Plasma progranulin levels predict progranulin mutation status in frontotemporal dementia patients and asymptomatic family members. Brain.

[32] Floris G, Borghero G, Cannas A, Di Stefano F, Murru MR, Corongiu D, Cuccu S, Tranquilli S, Cherchi MV, Serra A (2015). Clinical phenotypes and radiological findings in frontotemporal dementia related to TARDBP mutations. J Neurol.

[33] Freischmidt A, Wieland T, Richter B, Ruf W, Schaeffer V, Muller K, Marroquin N, Nordin F, Hubers A, Weydt P (2015). Haploinsufficiency of TBK1 causes familial ALS and fronto-temporal dementia. Nat Neurosci.

[34] Gabriel TL, Tol MJ, Ottenhof R, van Roomen C, Aten J, Claessen N, Hooibrink B, de Weijer B, Serlie MJ, Argmann C (2014). Lysosomal stress in obese adipose tissue macrophages contributes to MITF-dependent Gpnmb induction. Diabetes.

[35] Gass J, Lee WC, Cook C, Finch N, Stetler C, Jansen-West K, Lewis J, Link CD, Rademakers R, Nykjaer A (2012). Progranulin regulates neuronal outgrowth independent of sortilin. Mol Neurodegener.

[36] Ghidoni R, Benussi L, Glionna M, Franzoni M, Binetti G (2008). Low plasma progranulin levels predict progranulin mutations in frontotemporal lobar degeneration. Neurology.

[37] Ghoshal N, Dearborn JT, Wozniak DF, Cairns NJ (2012). Core features of frontotemporal dementia recapitulated in progranulin knockout mice. Neurobiol Dis.

[38] Gijselinck I, Van Mossevelde S, van der Zee J, Sieben A, Philtjens S, Heeman B, Engelborghs S, Vandenbulcke M, De Baets G, Baumer V (2015). Loss of TBK1 is a frequent cause of frontotemporal dementia in a Belgian cohort. Neurology.

[39] Goldman JS, Farmer JM, Wood EM, Johnson JK, Boxer A, Neuhaus J, Lomen-Hoerth C, Wilhelmsen KC, Lee VM, Grossman M (2005). Comparison of family histories in FTLD subtypes and related tauopathies. Neurology.

[40] Gossye H, Van Broeckhoven C, Engelborghs S (2019). The use of biomarkers and genetic screening to diagnose frontotemporal dementia: evidence and clinical implications. Front Neurosci.

[41] Götzl JK, Brendel M, Werner G, Parhizkar S, Sebastian Monasor L, Kleinberger G, Colombo AV, Deussing M, Wagner M, Winkelmann J (2019). Opposite microglial activation stages upon loss of PGRN or TREM2 result in reduced cerebral glucose metabolism. EMBO Mol Med.

[42] Gotzl JK, Colombo AV, Fellerer K, Reifschneider A, Werner G, Tahirovic S, Haass C, Capell A (2018). Early lysosomal maturation deficits in microglia triggers enhanced lysosomal activity in other brain cells of progranulin knockout mice. Mol Neurodegener.

[43] Greaves CV, Rohrer JD (2019). An update on genetic frontotemporal dementia. J Neurol.

[44] Guerreiro R, Gibbons E, Tabuas-Pereira M, Kun-Rodrigues C, Santo GC, Bras J (2020). Genetic architecture of common non-Alzheimer's disease dementias. Neurobiol Dis.

[45] Guyant-Marechal L, Laquerriere A, Duyckaerts C, Dumanchin C, Bou J, Dugny F, Le Ber I, Frebourg T, Hannequin D, Campion D (2006). Valosin-containing protein gene mutations: clinical and neuropathologic features. Neurology.

[46] Hendrickx DAE, van Scheppingen J, van der Poel M, Bossers K, Schuurman KG, van Eden CG, Hol EM, Hamann J, Huitinga I (2017). Gene expression profiling of multiple sclerosis pathology identifies early patterns of demyelination surrounding chronic active lesions. Front Immunol.

[47] Holler CJ, Taylor G, Deng Q, Kukar T (2017). Intracellular proteolysis of progranulin generates stable, lysosomal granulins that are haploinsufficient in patients with frontotemporal dementia caused by GRN mutations. eNeuro.

[48] Holler CJ, Taylor G, McEachin ZT, Deng Q, Watkins WJ, Hudson K, Easley CA, Hu WT, Hales CM, Rossoll W (2016). Trehalose upregulates progranulin expression in human and mouse models of GRN haploinsufficiency: a novel therapeutic lead to treat frontotemporal dementia. Mol Neurodegener.

[49] Horowitz AM, Fan X, Bieri G, Smith LK, Sanchez-Diaz CI, Schroer AB, Gontier G, Casaletto KB, Kramer JH, Williams KE (2020). Blood factors transfer beneficial effects of exercise on neurogenesis and cognition to the aged brain. Science.

[50] Huin V, Barbier M, Bottani A, Lobrinus JA, Clot F, Lamari F, Chat L, Rucheton B, Fluchere F, Auvin S (2020). Homozygous GRN mutations: new phenotypes and new insights into pathological and molecular mechanisms. Brain.

[51] Huttenrauch M, Ogorek I, Klafki H, Otto M, Stadelmann C, Weggen S, Wiltfang J, Wirths O (2018). Glycoprotein NMB: a novel Alzheimer's disease associated marker expressed in a subset of activated microglia. Acta Neuropathol Commun.

[52] International Parkinson's Disease Genomics C, Wellcome Trust Case Control C (2011). A two-stage meta-analysis identifies several new loci for Parkinson's disease. PLoS Genet.

[53] Johnson ECB, Dammer EB, Duong DM, Ping L, Zhou M, Yin L, Higginbotham LA, Guajardo A, White B, Troncoso JC (2020). Large-scale proteomic analysis of Alzheimer's disease brain and cerebrospinal fluid reveals early changes in energy metabolism associated with microglia and astrocyte activation. Nat Med.

[54] Josephs KA, Zhang YJ, Baker M, Rademakers R, Petrucelli L, Dickson DW (2019). C-terminal and full length TDP-43 specie differ according to FTLD-TDP lesion type but not genetic mutation. Acta Neuropathol Commun.

[55] Jung JI, Ran Y, Cruz PE, Rosario AM, Ladd TB, Kukar TL, Koo EH, Felsenstein KM, Golde TE (2014). Complex relationships between substrate sequence and sensitivity to alterations in gamma-secretase processivity induced by gamma-secretase modulators. Biochemistry.

[56] Klein AD, Mazzulli JR (2018). Is Parkinson's disease a lysosomal disorder?. Brain.

[57] Kleinberger G, Capell A, Haass C, Van Broeckhoven C (2013). Mechanisms of granulin deficiency: lessons from cellular and animal models. Mol Neurobiol.

[58] Klionsky DJ, Abdelmohsen K, Abe A, Abedin MJ, Abeliovich H, Acevedo Arozena A, Adachi H, Adams CM, Adams PD, Adeli K (2016). Guidelines for the use and interpretation of assays for monitoring autophagy (3rd edition). Autophagy.

[59] Koster KP, Yoshii A (2019). Depalmitoylation by palmitoyl-protein thioesterase 1 in neuronal health and degeneration. Front Synaptic Neurosci.

[60] Krabbe G, Minami SS, Etchegaray JI, Taneja P, Djukic B, Davalos D, Le D, Lo I, Zhan L, Reichert MC (2017). Microglial NFkappaB-TNFalpha hyperactivation induces obsessive-compulsive behavior in mouse models of progranulin-deficient frontotemporal dementia. Proc Natl Acad Sci USA.

[61] Kramer G, Wegdam W, Donker-Koopman W, Ottenhoff R, Gaspar P, Verhoek M, Nelson J, Gabriel T, Kallemeijn W, Boot RG (2016). Elevation of glycoprotein nonmetastatic melanoma protein B in type 1 Gaucher disease patients and mouse models. FEBS Open Bio.

[62] Krasemann S, Madore C, Cialic R, Baufeld C, Calcagno N, El Fatimy R, Beckers L, O'Loughlin E, Xu Y, Fanek Z (2017). The TREM2-APOE pathway drives the transcriptional phenotype of dysfunctional microglia in neurodegenerative diseases. Immunity.

[63] Kukar T, Murphy MP, Eriksen JL, Sagi SA, Weggen S, Smith TE, Ladd T, Khan MA, Kache R, Beard J (2005). Diverse compounds mimic Alzheimer disease-causing mutations by augmenting Abeta42 production. Nat Med.

[64] Kukar TL, Ladd TB, Bann MA, Fraering PC, Narlawar R, Maharvi GM, Healy B, Chapman R, Welzel AT, Price RW (2008). Substrate-targeting gamma-secretase modulators. Nature.

[65] Laird AS, Van Hoecke A, De Muynck L, Timmers M, Van den Bosch L, Van Damme P, Robberecht W (2010). Progranulin is neurotrophic in vivo and protects against a mutant TDP-43 induced axonopathy. PLoS ONE.

[66] Langfelder P, Horvath S (2008). WGCNA: an R package for weighted correlation network analysis. BMC Bioinform.

[67] Le Ber I, Camuzat A, Guerreiro R, Bouya-Ahmed K, Bras J, Nicolas G, Gabelle A, Didic M, De Septenville A, Millecamps S (2013). SQSTM1 mutations in French patients with frontotemporal dementia or frontotemporal dementia with amyotrophic lateral sclerosis. JAMA Neurol.

[68] Lee CW, Stankowski JN, Chew J, Cook CN, Lam YW, Almeida S, Carlomagno Y, Lau KF, Prudencio M, Gao FB (2017). The lysosomal protein cathepsin L is a progranulin protease. Mol Neurodegener.

[69] Li Q, Cheng Z, Zhou L, Darmanis S, Neff NF, Okamoto J, Gulati G, Bennett ML, Sun LO, Clarke LE (2019). Developmental heterogeneity of microglia and brain myeloid cells revealed by deep single-cell RNA sequencing. Neuron.

[70] Lui H, Zhang J, Makinson SR, Cahill MK, Kelley KW, Huang HY, Shang Y, Oldham MC, Martens LH, Gao F (2016). Progranulin deficiency promotes circuit-specific synaptic pruning by microglia via complement activation. Cell.

[71] Marshall J, Nietupski JB, Park H, Cao J, Bangari DS, Silvescu C, Wilper T, Randall K, Tietz D, Wang B (2019). Substrate reduction therapy for sandhoff disease through inhibition of glucosylceramide synthase activity. Mol Ther.

[72] McEachin ZT, Gendron TF, Raj N, Garcia-Murias M, Banerjee A, Purcell RH, Ward PJ, Todd TW, Merritt-Garza ME, Jansen-West K (2020). Chimeric peptide species contribute to divergent dipeptide repeat pathology in c9ALS/FTD and SCA36. Neuron.

[73] Meeter LH, Patzke H, Loewen G, Dopper EG, Pijnenburg YA, van Minkelen R, van Swieten JC (2016). Progranulin levels in plasma and cerebrospinal fluid in granulin mutation carriers. Dement Geriatr Cogn Dis Extra.

[74] Miller JA, Woltjer RL, Goodenbour JM, Horvath S, Geschwind DH (2013). Genes and pathways underlying regional and cell type changes in Alzheimer's disease. Genome Med.

[75] Moloney EB, Moskites A, Ferrari EJ, Isacson O, Hallett PJ (2018). The glycoprotein GPNMB is selectively elevated in the substantia nigra of Parkinson's disease patients and increases after lysosomal stress. Neurobiol Dis.

[76] Monami G, Gonzalez EM, Hellman M, Gomella LG, Baffa R, Iozzo RV, Morrione A (2006). Proepithelin promotes migration and invasion of 5637 bladder cancer cells through the activation of ERK1/2 and the formation of a paxillin/FAK/ERK complex. Cancer Res.

[77] Moore BD, Martin J, de Mena L, Sanchez J, Cruz PE, Ceballos-Diaz C, Ladd TB, Ran Y, Levites Y, Kukar TL (2018). Short Abeta peptides attenuate Abeta42 toxicity in vivo. J Exp Med.

[78] Murugesan V, Liu J, Yang R, Lin H, Lischuk A, Pastores G, Zhang X, Chuang WL, Mistry PK (2018). Validating glycoprotein non-metastatic melanoma B (gpNMB, osteoactivin), a new biomarker of Gaucher disease. Blood Cells Mol Dis.

[79] Nagahara Y, Shimazawa M, Ohuchi K, Ito J, Takahashi H, Tsuruma K, Kakita A, Hara H (2017). GPNMB ameliorates mutant TDP-43-induced motor neuron cell death. J Neurosci Res.

[80] Neill T, Buraschi S, Goyal A, Sharpe C, Natkanski E, Schaefer L, Morrione A, Iozzo RV (2016). EphA2 is a functional receptor for the growth factor progranulin. J Cell Biol.

[81] Neumann M, Sampathu DM, Kwong LK, Truax AC, Micsenyi MC, Chou TT, Bruce J, Schuck T, Grossman M, Clark CM (2006). Ubiquitinated TDP-43 in frontotemporal lobar degeneration and amyotrophic lateral sclerosis. Science.

[82] Nguyen AD, Nguyen TA, Zhang J, Devireddy S, Zhou P, Karydas AM, Xu X, Miller BL, Rigo F, Ferguson SM (2018). Murine knockin model for progranulin-deficient frontotemporal dementia with nonsense-mediated mRNA decay. Proc Natl Acad Sci USA.

[83] Nguyen M, Wong YC, Ysselstein D, Severino A, Krainc D (2019). Synaptic, mitochondrial, and lysosomal dysfunction in Parkinson's disease. Trends Neurosci.

[84] Oldham MC, Konopka G, Iwamoto K, Langfelder P, Kato T, Horvath S, Geschwind DH (2008). Functional organization of the transcriptome in human brain. Nat Neurosci.

[85] Palfree RG, Bennett HP, Bateman A (2015). The evolution of the secreted regulatory protein progranulin. PLoS ONE.

[86] Panza F, Lozupone M, Seripa D, Daniele A, Watling M, Giannelli G, Imbimbo BP (2020). Development of disease-modifying drugs for frontotemporal dementia spectrum disorders. Nat Rev Neurol.

[87] Parveen F, Bender D, Law SH, Mishra VK, Chen CC, Ke LY (2019). Role of ceramidases in sphingolipid metabolism and human diseases. Cells.

[88] Petkau TL, Neal SJ, Orban PC, MacDonald JL, Hill AM, Lu G, Feldman HH, Mackenzie IR, Leavitt BR (2010). Progranulin expression in the developing and adult murine brain. J Comp Neurol.

[89] Pihlstrom L, Axelsson G, Bjornara KA, Dizdar N, Fardell C, Forsgren L, Holmberg B, Larsen JP, Linder J, Nissbrandt H (2013). Supportive evidence for 11 loci from genome-wide association studies in Parkinson's disease. Neurobiol Aging.

[90] Ping L, Duong DM, Yin L, Gearing M, Lah JJ, Levey AI, Seyfried NT (2018). Global quantitative analysis of the human brain proteome in Alzheimer's and Parkinson's disease. Sci Data.

[91] Platt FM, d'Azzo A, Davidson BL, Neufeld EF, Tifft CJ (2018). Lysosomal storage diseases. Nat Rev Dis Primers.

[92] Pottier C, Bieniek KF, Finch N, van de Vorst M, Baker M, Perkersen R, Brown P, Ravenscroft T, van Blitterswijk M, Nicholson AM (2015). Whole-genome sequencing reveals important role for TBK1 and OPTN mutations in frontotemporal lobar degeneration without motor neuron disease. Acta Neuropathol.

[93] Raben N, Puertollano R (2016). TFEB and TFE3: linking lysosomes to cellular adaptation to stress. Annu Rev Cell Dev Biol.

[94] Ramms B, Gordts P (2018). Apolipoprotein C-III in triglyceride-rich lipoprotein metabolism. Curr Opin Lipidol.

[95] Ramos EM, Dokuru DR, Van Berlo V, Wojta K, Wang Q, Huang AY, Deverasetty S, Qin Y, van Blitterswijk M, Jackson J (2020). Genetic screening of a large series of North American sporadic and familial frontotemporal dementia cases. Alzheimers Dement.

[96] Roberson ED (2012). Mouse models of frontotemporal dementia. Ann Neurol.

[97] Rosen HJ, Boeve BF, Boxer AL (2020). Tracking disease progression in familial and sporadic frontotemporal lobar degeneration: recent findings from ARTFL and LEFFTDS. Alzheimers Dement.

[98] Sardiello M, Palmieri M, di Ronza A, Medina DL, Valenza M, Gennarino VA, Di Malta C, Donaudy F, Embrione V, Polishchuk RS (2009). A gene network regulating lysosomal biogenesis and function. Science.

[99] Sarkar S, Dammer EB, Malovic E, Olsen AL, Raza SA, Gao T, Xiao H, Oliver DL, Duong D, Joers V (2020). Molecular signatures of neuroinflammation induced by alphasynuclein aggregates in microglial cells. Front Immunol.

[100] Satoh JI, Kino Y, Yanaizu M, Ishida T, Saito Y (2019). Microglia express GPNMB in the brains of Alzheimer's disease and Nasu-Hakola disease. Intractable Rare Dis Res.

[101] Settembre C, Di Malta C, Polito VA, Garcia Arencibia M, Vetrini F, Erdin S, Erdin SU, Huynh T, Medina D, Colella P (2011). TFEB links autophagy to lysosomal biogenesis. Science.

[102] Seyfried NT, Dammer EB, Swarup V, Nandakumar D, Duong DM, Yin L, Deng Q, Nguyen T, Hales CM, Wingo T (2017). A multi-network approach identifies protein-specific co-expression in asymptomatic and symptomatic Alzheimer's disease. Cell Syst.

[103] Sharma K, Schmitt S, Bergner CG, Tyanova S, Kannaiyan N, Manrique-Hoyos N, Kongi K, Cantuti L, Hanisch UK, Philips MA (2015). Cell type- and brain region-resolved mouse brain proteome. Nat Neurosci.

[104] Skibinski G, Parkinson NJ, Brown JM, Chakrabarti L, Lloyd SL, Hummerich H, Nielsen JE, Hodges JR, Spillantini MG, Thusgaard T (2005). Mutations in the endosomal ESCRTIII-complex subunit CHMP2B in frontotemporal dementia. Nat Genet.

[105] Smith KR, Damiano J, Franceschetti S, Carpenter S, Canafoglia L, Morbin M, Rossi G, Pareyson D, Mole SE, Staropoli JF (2012). Strikingly different clinicopathological phenotypes determined by progranulin-mutation dosage. Am J Hum Genet.

[106] Song L, Langfelder P, Horvath S (2012). Comparison of co-expression measures: mutual information, correlation, and model based indices. BMC Bioinform.

[107] Sudre CH, Bocchetta M, Heller C, Convery R, Neason M, Moore KM, Cash DM, Thomas DL, Woollacott IOC, Foiani M (2019). White matter hyperintensities in progranulin-associated frontotemporal dementia: a longitudinal GENFI study. Neuroimage Clin.

[108] Tanaka Y, Chambers JK, Matsuwaki T, Yamanouchi K, Nishihara M (2014). Possible involvement of lysosomal dysfunction in pathological changes of the brain in aged progranulin-deficient mice. Acta Neuropathol Commun.

[109] Thomas L, Pasquini LA (2018). Galectin-3-mediated glial crosstalk drives oligodendrocyte differentiation and (re)myelination. Front Cell Neurosci.

[110] Thygesen C, Ilkjaer L, Kempf SJ, Hemdrup AL, von Linstow CU, Babcock AA, Darvesh S, Larsen MR, Finsen B (2018). Diverse protein profiles in CNS myeloid cells and CNS tissue from lipopolysaccharide- and vehicle-injected APPSWE/PS1DeltaE9 transgenic mice implicate cathepsin Z in Alzheimer's disease. Front Cell Neurosci.

[111] Tol MJ, van der Lienden MJC, Gabriel TL, Hagen JJ, Scheij S, Veenendaal T, Klumperman J, Donker-Koopman WE, Verhoeven AJ, Overkleeft H (2018). HEPES activates a MiT/TFE-dependent lysosomal-autophagic gene network in cultured cells: a call for caution. Autophagy.

[112] Tolkatchev D, Malik S, Vinogradova A, Wang P, Chen Z, Xu P, Bennett HP, Bateman A, Ni F (2008). Structure dissection of human progranulin identifies well-folded granulin/epithelin modules with unique functional activities. Protein Sci.

[113] Tsou PS, Sawalha AH (2020). Glycoprotein nonmetastatic melanoma protein B: a key mediator and an emerging therapeutic target in autoimmune diseases. FASEB J.

[114] Tsuboi K, Sun YX, Okamoto Y, Araki N, Tonai T, Ueda N (2005). Molecular characterization of N-acylethanolamine-hydrolyzing acid amidase, a novel member of the choloylglycine hydrolase family with structural and functional similarity to acid ceramidase. J Biol Chem.

[115] Valdez C, Wong YC, Schwake M, Bu G, Wszolek ZK, Krainc D (2017). Progranulin-mediated deficiency of cathepsin D results in FTD and NCL-like phenotypes in neurons derived from FTD patients. Hum Mol Genet.

[116] Valdez C, Ysselstein D, Young TJ, Zheng J, Krainc D (2020). Progranulin mutations result in impaired processing of prosaposin and reduced glucocerebrosidase activity. Hum Mol Genet.

[117] Van Damme P, Van Hoecke A, Lambrechts D, Vanacker P, Bogaert E, van Swieten J, Carmeliet P, Van Den Bosch L, Robberecht W (2008). Progranulin functions as a neurotrophic factor to regulate neurite outgrowth and enhance neuronal survival. J Cell Biol.

[118] van der Ende EL, Meeter LH, Poos JM, Panman JL, Jiskoot LC, Dopper EGP, Papma JM, de Jong FJ, Verberk IMW, Teunissen C (2019). Serum neurofilament light chain in genetic frontotemporal dementia: a longitudinal, multicentre cohort study. Lancet Neurol.

[119] van der Lienden MJC, Gaspar P, Boot R, Aerts J, van Eijk M (2018). Glycoprotein non-metastatic protein B: an emerging biomarker for lysosomal dysfunction in macrophages. Int J Mol Sci.

[120] van der Zee J, Urwin H, Engelborghs S, Bruyland M, Vandenberghe R, Dermaut B, De Pooter T, Peeters K, Santens P, De Deyn PP (2008). CHMP2B C-truncating mutations in frontotemporal lobar degeneration are associated with an aberrant endosomal phenotype in vitro. Hum Mol Genet.

[121] van der Zee J, Van Langenhove T, Kovacs GG, Dillen L, Deschamps W, Engelborghs S, Matej R, Vandenbulcke M, Sieben A, Dermaut B (2014). Rare mutations in SQSTM1 modify susceptibility to frontotemporal lobar degeneration. Acta Neuropathol.

[122] Verbeeck C, Deng Q, Dejesus-Hernandez M, Taylor G, Ceballos-Diaz C, Kocerha J, Golde T, Das P, Rademakers R, Dickson DW (2012). Expression of Fused in sarcoma mutations in mice recapitulates the neuropathology of FUS proteinopathies and provides insight into disease pathogenesis. Mol Neurodegener.

[123] Wallings RL, Humble SW, Ward ME, Wade-Martins R (2019). Lysosomal dysfunction at the centre of Parkinson's disease and frontotemporal dementia/amyotrophic lateral sclerosis. Trends Neurosci.

[124] Wang SY, Chen W, Xu W, Li JQ, Hou XH, Ou YN, Yu JT, Tan L (2019). Neurofilament light chain in cerebrospinal fluid and blood as a biomarker for neurodegenerative diseases: a systematic review and meta-analysis. J Alzheimers Dis.

[125] Ward ME, Chen R, Huang HY, Ludwig C, Telpoukhovskaia M, Taubes A, Boudin H, Minami SS, Reichert M, Albrecht P (2017). Individuals with progranulin haploinsufficiency exhibit features of neuronal ceroid lipofuscinosis. Sci Transl Med.

[126] Watts GD, Wymer J, Kovach MJ, Mehta SG, Mumm S, Darvish D, Pestronk A, Whyte MP, Kimonis VE (2004). Inclusion body myopathy associated with Paget disease of bone and frontotemporal dementia is caused by mutant valosin-containing protein. Nat Genet.

[127] Wils H, Kleinberger G, Pereson S, Janssens J, Capell A, Van Dam D, Cuijt I, Joris G, De Deyn PP, Haass C (2012). Cellular ageing, increased mortality and FTLD-TDP-associated neuropathology in progranulin knockout mice. J Pathol.

[128] Woollacott IOC, Bocchetta M, Sudre CH, Ridha BH, Strand C, Courtney R, Ourselin S, Cardoso MJ, Warren JD, Rossor MN (2018). Pathological correlates of white matter hyperintensities in a case of progranulin mutation associated frontotemporal dementia. Neurocase.

[129] Xia X, Serrero G (1998). Identification of cell surface binding sites for PC-cell-derived growth factor, PCDGF, (epithelin/granulin precursor) on epithelial cells and fibroblasts. Biochem Biophys Res Commun.

[130] Xu J, Xilouri M, Bruban J, Shioi J, Shao Z, Papazoglou I, Vekrellis K, Robakis NK (2011). Extracellular progranulin protects cortical neurons from toxic insults by activating survival signaling. Neurobiol Aging.

[131] Yin F, Banerjee R, Thomas B, Zhou P, Qian L, Jia T, Ma X, Ma Y, Iadecola C, Beal MF (2010). Exaggerated inflammation, impaired host defense, and neuropathology in progranulin-deficient mice. J Exp Med.

[132] Yin F, Dumont M, Banerjee R, Ma Y, Li H, Lin MT, Beal MF, Nathan C, Thomas B, Ding A (2010). Behavioral deficits and progressive neuropathology in progranulin-deficient mice: a mouse model of frontotemporal dementia. FASEB J.

[133] Zhou X, Paushter DH, Feng T, Sun L, Reinheckel T, Hu F (2017). Lysosomal processing of progranulin. Mol Neurodegener.

[134] Zhou X, Paushter DH, Pagan MD, Kim D, Nunez Santos M, Lieberman RL, Overkleeft HS, Sun Y, Smolka MB, Hu F (2019). Progranulin deficiency leads to reduced glucocerebrosidase activity. PLoS ONE.

[135] Zhou X, Sun L, Bracko O, Choi JW, Jia Y, Nana AL, Brady OA, Hernandez JCC, Nishimura N, Seeley WW (2017). Impaired prosaposin lysosomal trafficking in frontotemporal lobar degeneration due to progranulin mutations. Nat Commun.

[136] Zhou Y, Zhou B, Pache L, Chang M, Khodabakhshi AH, Tanaseichuk O, Benner C, Chanda SK (2019). Metascape provides a biologist-oriented resource for the analysis of systems-level datasets. Nat Commun.

[137] Zhu J, Nathan C, Jin W, Sim D, Ashcroft GS, Wahl SM, Lacomis L, Erdjument-Bromage H, Tempst P, Wright CD (2002). Conversion of proepithelin to epithelins: roles of SLPI and elastase in host defense and wound repair. Cell.

[138] Zigdon H, Savidor A, Levin Y, Meshcheriakova A, Schiffmann R, Futerman AH (2015). Identification of a biomarker in cerebrospinal fluid for neuronopathic forms of Gaucher disease. PLoS ONE.

