# Supplementary material for: Network analysis of the progranulin-deficient mouse brain proteome reveals pathogenic mechanisms shared in human frontotemporal dementia caused by GRN mutations
Source: Acta Neuropathol Commun. 2020 Oct 7;8:163. doi: 10.1186/s40478-020-01037-x (PMC7541308; doi:10.1186/s40478-020-01037-x)
Supplement: Supplementary file 2 — Additional file 2. Table S2 summarizing the neuropathological, clinical diagnosis, age, sex, and other characterizes of human post-mortem samples used for immunostaining and ELISA. [file 40478_2020_1037_MOESM2_ESM.docx]

**Table S2:**

Neuropathological and Clinical data for samples from Emory Brain Tissue Bank (Emory University Goizueta Alzheimer’s Disease Research Center, Atlanta, Georgia) and Mayo Clinic Brain Bank (Jacksonville, Florida).

Demographic data and characteristics of post-mortem samples used in ELISA quantifications.

|  | **FTD-*GRN*** | **Cognitively Normal Controls** |
| --- | --- | --- |
| Number | 21 | 23 |
| Sex (M/F) | 11/10 | 13/10 |
| Age (y) | 66.81± 1.66 | 62.17± 2.51 |

| **ID** | **PathDx** | **Mutation** | **Sex** | | **Age** | **Braak Stage** | **Thal phase** | **Clinical Dx** | **FHx** |
| --- | --- | --- | --- | --- | --- | --- | --- | --- | --- |
| OS03-27 | FTLD-TDP | GRN | M | 63 | | 0 | 2 | FTD | Y |
| E05-108 | FTLD-TDP | GRN | F | 62 | | 0 | 0 | FTD | Y |
| E07-48 | FTLD-TDP | GRN | M | 71 | | 1 | 2 | AD | Y |
| E10-38 | FTLD-TDP | GRN | M | 62 | | 1 | 2 | CBD | Y |
| E11-128 | FTLD-TDP | GRN | M | 63 | | 1 | 0 | PPA | Y |
| E14-53 | FTLD-TDP | GRN | F | 61 | | 3 | 1 | FTD | Y |
| E16-16 | FTLD-TDP | GRN | F | 61 | | 1 | 2 | FTD-*GRN* | Y |
| MC2 | FTLD-TDP | GRN | F | 81 | | 0 | 0 | None | Y |
| MC4 | FTLD-TDP | GRN | F | 63 | | 0 | 0 | FTD/CBD | Y |
| MC9 | FTLD-TDP | GRN | M | 66 | | 0 | 0 | PNFA | Y |
| MC14 | FTLD-TDP | GRN | M | 56 | | 0 | 1 | AD | Y |
| MC15 | FTLD-TDP | GRN | F | 75 | | 0 | 1 | PiD | Y |
| MC16 | FTLD-TDP | GRN | F | 59 | | 1 | 0 | PNFA | Y |
| MC19 | FTLD-TDP | GRN | F | 66 | | 0 | 0 | AD | Y |
| MC20 | FTLD-TDP | GRN | M | 73 | | 0 | 1 | PNFA | Y |
| MC22 | FTLD-TDP | GRN | M | 87 | | I | 0 | AD | Y |
| MC23 | FTLD-TDP | GRN | F | 75 | | 0 | 0 | PiD | Y |
| MC24 | FTLD-TDP | GRN | M | 63 | | 0 | 0 | FTD | Y |
| MC25 | FTLD-TDP | GRN | M | 64 | | 0 | 0 | PNFA | Y |
| MC27 | FTLD-TDP | GRN | M | 65 | | 0 | 0 | CBD | Y |
| MC29 | FTLD-TDP | GRN | F | 67 | | 0 | 0 | FTD-*GRN* | Y |
| A86-46 | Normal | - | M | 65 | | 1 | 1 | Lung Cancer | Unk |
| A93-03 | Normal | - | M | 70 | | 1 | 2 | Pelvis Cancer; hx abdominal aortic aneurysms - one repaired, one unruptured | N |
| OS00-06 | Normal | - | F | 60 | | 1 | 1 | Diabetes, COPD, Renal Insufficiency | Unk |
| OS03-299 | Normal | - | M | 69 | | 2 | 2 | COPD, Lung cancer | Unk |
| OS03-380 | Normal | - | M | 61 | | 2 | 2 | Diabetes, Renal disease, Congestive heart failure | Unk |
| E04-34 | Normal | - | F | 57 | | 1 | 2 | Aortic aneurysm | Unk |
| E05-74 | Normal | - | M | 59 | | 1 | 0 | Ischemic heart disease and fibrosis | Unk |
| E06-113 | Normal | - | F | 20 | | 0 | 0 | Aplastic anemia | Unk |
| 101 | Normal | - | F | 55 | | 0 | 0 | CJD | N |
| 102 | Normal | - | M | 78 | | 2 | 1 | Depression | N |
| 103 | Normal | - | F | 74 | | 3 | 0 | NCI | N |
| 104 | Normal | - | F | 56 | | 0 | 0 | Normal | N |
| 105 | Normal | - | M | 69 | | 2 | 0 | AD v DLB | N |
| 106 | Normal | - | F | 64 | | 1 | 1 | Dysautonomia | N |
| 107 | Normal | - | M | 75 | | 3 | 0 | PRKN kindred | N |
| 108 | Normal | - | M | 63 | | 3 | 0 | DLB v FTD | N |
| 109 | Normal | - | F | 60 | | 1 | 1 | NAIM | N |
| 110 | Normal | - | M | 53 | | 1 | 0 | FTD | N |
| 111 | Normal | - | F | 56 | | 1 | 0 | PSP/PLS/schizoaffective | Y |
| 112 | Normal | - | M | 61 | | 1 | 0 | Torsion dystonia (s/p DBS) | N |
| 113 | Normal | - | M | 78 | | 2 | 0 | DLB | N |
| 114 | Normal | - | F | 54 | | 1 | 0 | Liposarcoma/Tx-related Neuropathy | N |
| 115 | Normal | - | M | 73 | | 1 | 0 | Ulcerative colitis | N |

Characteristics of individuals used in immunohistochemistry staining.

|  | **ID** | **PathDx** | | **Mutation** | | **Sex** | | **Age** | | **Braak Stage** | | **Thal phase** | | **Clinical Dx** | | **FHx** | |
| --- | --- | --- | --- | --- | --- | --- | --- | --- | --- | --- | --- | --- | --- | --- | --- | --- | --- |
| P1 | E16-16 | FTLD-TDP | GRN | | F | | 61 | | 1 | | 2 | | FTD-*GRN* | | Y | |  |
| P2 | OS03-27 | FTLD-TDP | GRN | | M | | 63 | | 0 | | 2 | | FTD | | Y | |  |
| P3 | E07-48 | FTLD-TDP | GRN | | M | | 71 | | 1 | | 2 | | AD | | Y | |  |
| P4 | E05-108 | FTLD-TDP | GRN | | F | | 62 | | 0 | | 0 | | FTD | | Y | |  |
| P5 | E14-53 | FTLD-TDP | GRN | | F | | 61 | | 3 | | 1 | | FTD | | Y | |  |
| C1 | OS00-06 | Normal | - | | F | | 60 | | 1 | | 1 | | Diabetes, COPD, Renal Insufficiency | | Unk | |  |
| C2 | OS03-299 | Normal | - | | M | | 69 | | 2 | | 2 | | COPD, Lung cancer | | Unk | |  |
| C3 | OS03-380 | Normal | - | | M | | 61 | | 2 | | 2 | | Diabetes, Renal disease, Congestive heart failure | | Unk | |  |
| C4 | E04-34 | Normal | - | | F | | 57 | | 1 | | 2 | | Aortic aneurysm | | Unk | |  |
| C5 | E05-74 | Normal | - | | M | | 59 | | 1 | | 0 | | Ischemic heart disease and fibrosis | | Unk | |  |

FHx: Familial History; P: Patient; C: Cognitively normal control

AD: Alzheimer’s Disease

CBD: Corticobasal degeneration

CJD: Creutzfeldt-Jakob Disease

COPD: Chronic Obstructive Pulmonary Disease

DLB: Dementia with Lewy Bodies

FTD: Frontotempoal Dementia

NAIM: Novaculitic Autoimmune Inflammatory Meningoencephalitis

NCI: Neuronal Cytoplasmic Inclusions

PiD: Pick's Disease

PLS: Primary Lateral Sclerosis

PNFA: Progressive nonfluent aphasia

PNA: Progressive nonfluent aphasia

PPA: Primary Progressive Aphasia

PSP: Progressive Supranuclear Palsy
