# Supplementary material for: Network analysis of the progranulin-deficient mouse brain proteome reveals pathogenic mechanisms shared in human frontotemporal dementia caused by GRN mutations
Source: Acta Neuropathol Commun. 2020 Oct 7;8:163. doi: 10.1186/s40478-020-01037-x (PMC7541308; doi:10.1186/s40478-020-01037-x)
Supplement: Supplementary file 3 — Additional file 3. Compilation of multiple supplementary figures (S1–S7). Fig. S1 is a two-color heatmap showing the relationship between WGCNA modules and the bicor correlation of age. Fig. S2 contains multiple Box plots of WGCNA modules (M1, M2, M4, M8, M9, M10, M11, M12, M13, M14, M15, M17, M18, M20, M21, M22, M23, M24, M25, and M26). Fig. S3 displays Igraphs of modules (M3, M5, M6, M7, M16, and M19) that are associated with knockout (Grn−/−) status and corresponding gene symbols as nodes. Fig. S4 displays Igraphs of modules (M1, M2, M4, M8, M9, M10, M11, M12, M13, M14, M15, M17, M18, M20, M21, M22, M23, M24, M25, and M26 modules) associated with knockout (Grn−/−) status and corresponding gene symbols as nodes. Fig. S5 summarizes the gene ontology (GO) enrichment analysis of proteins in M1, M2, M4, M8, M9, M10, M11, M12, M13, M14, M15, M17, M18, M20, M21, M22, M23, M24, M25, and M26 modules. Light-green bars: biological process, light-blue bars: molecular function, brown-bars: cellular component. Fig. S6 is an immunoadsorption validation experiment demonstrating an anti-human GPNMB antibody binds antigen specifically on human brain sections. Fig. S7 GPNMB immunostaining was performed on frontal lobe tissue sections from four FTD-GRN patients. [file 40478_2020_1037_MOESM3_ESM.pdf]

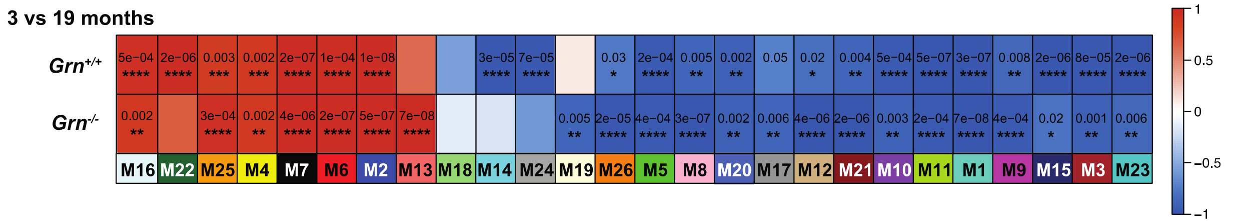

Supplementary Fig. S1 Two-color heatmap showing relationship between modules and the bicor correlation of age. Significance levels are **** p<0.0001, *** p <0.001, ** p <0.01 and * p < 0.05.

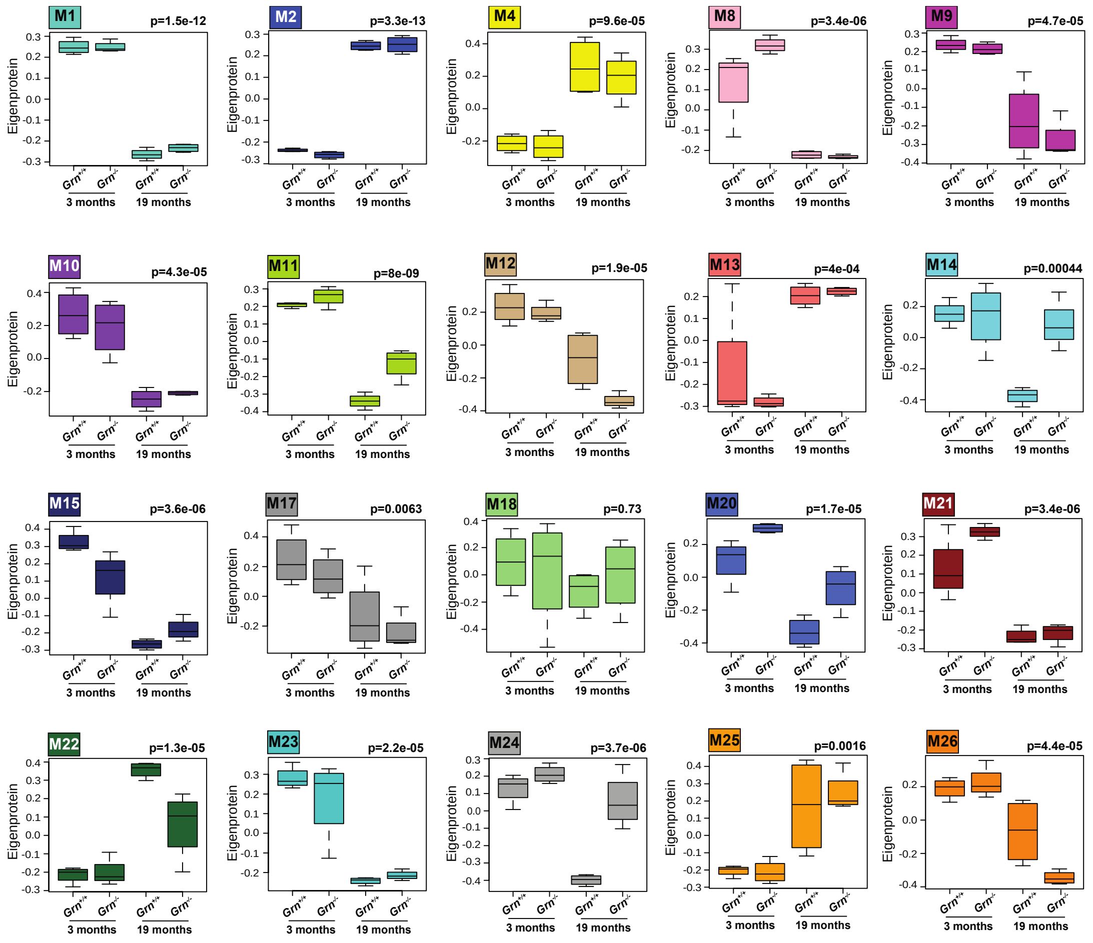

Supplementary Fig. S2 Box plots of M1, M2, M4, M8, M9, M10, M11, M12, M13, M14, M15, M17, M18, M20, M21, M22, M23, M24, M25, and M26 modules. Values were analyzed by two-way ANOVA.

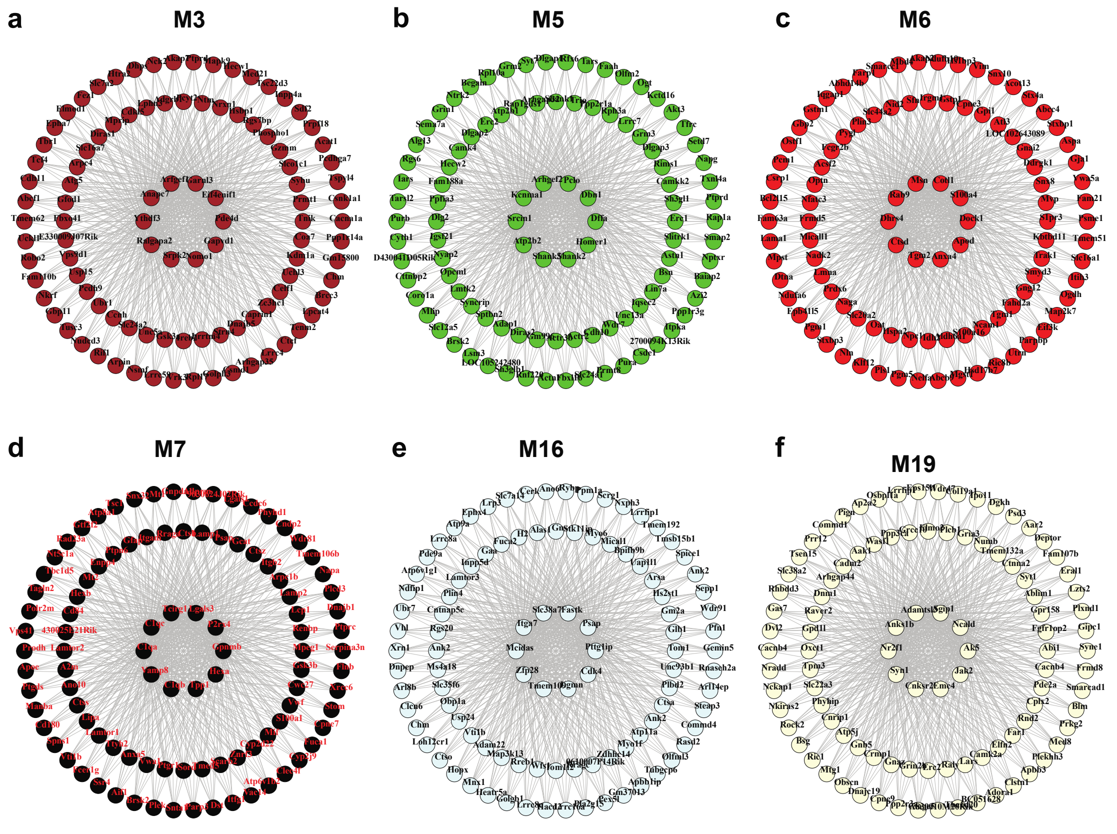

Supplementary Fig.S3 a-f Igraph of modules (M3, M5, M6, M7, M16, and M19) associated with knockout status and corresponding gene symbols as nodes. Node size and edges (gray) are reflective of the degree of intramodular connectivity (kME).

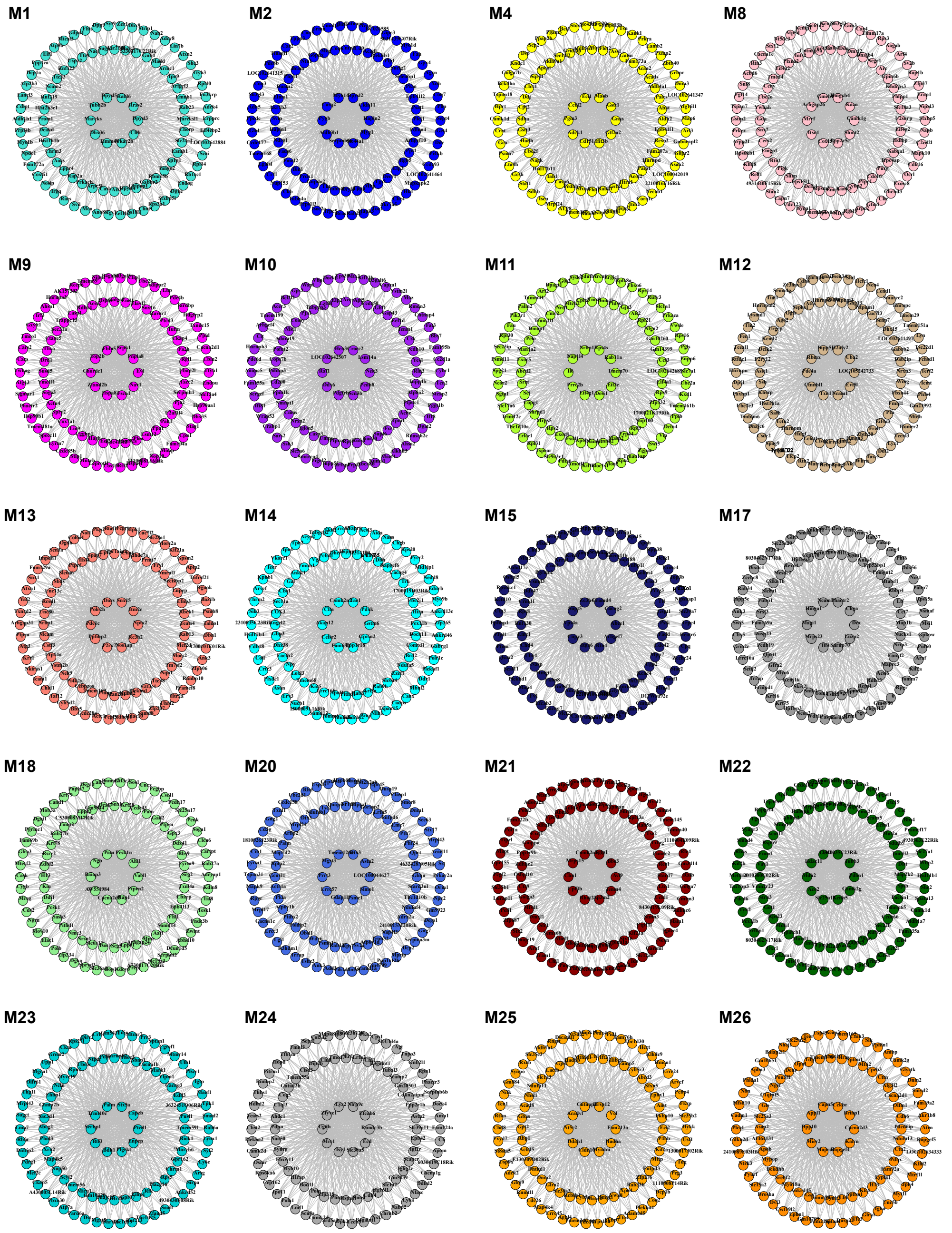

Supplementary Fig.S4 Igraph of modules (M1, M2, M4, M8, M9, M10, M11, M12, M13, M14, M15, M17, M18, M20, M21, M22, M23, M24, M25, and M26 modules) associated with knockout status and corresponding gene symbols as nodes. Node size and edges (gray) are reflective of the degree of intramodular connectivity (kME).

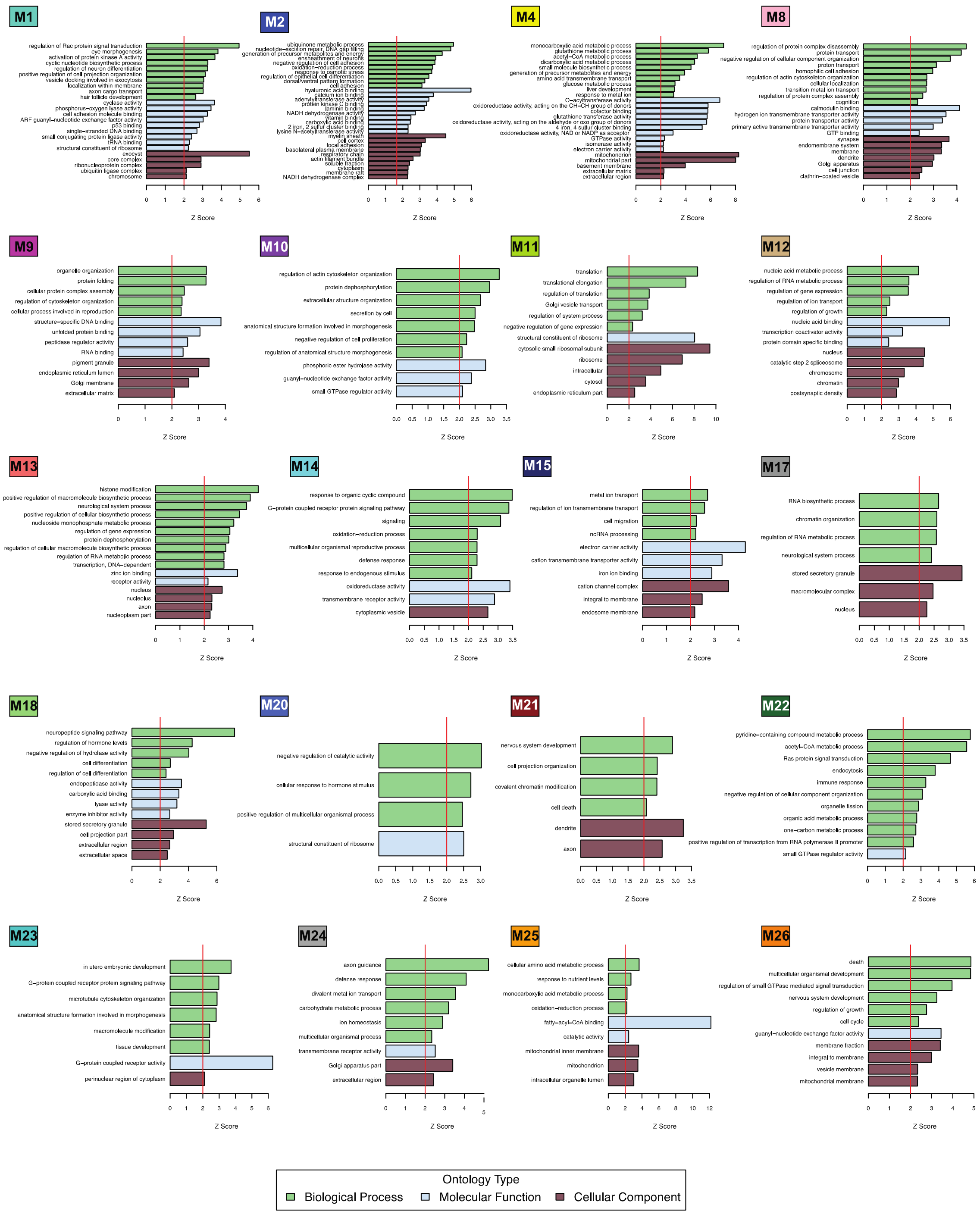

Supplementary Fig.S5 Gene ontology (GO) enrichment analysis of proteins in M1, M2, M4, M8, M9, M10, M11, M12, M13, M14, M15, M17, M18, M20, M21, M22, M23, M24, M25, and M26 modules. Light-green bars: biological process, light-blue bars: molecular function, brown-bars: cellular component.

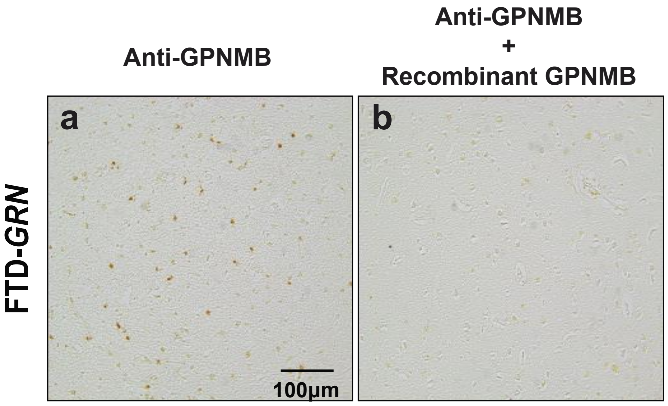

Supplementary Fig. S6 Immunoadsorption validation experiment demonstrates anti-human GPNMB antibody binds antigen specifically on human brain section. **a** Anti-human GPNMB antibody detected abundant GPNMB signals in frontal lobe section from FTD-*GRN* patient. **b** Addition of 2µg of recombinant human GPNMB to incubation buffer completely neutralized anti-human GPNMB antibody and no immunoreactivity signal was detected in frontal lobe section from FTD-*GRN* patient. Scale bars (100 µm) labeled in images.

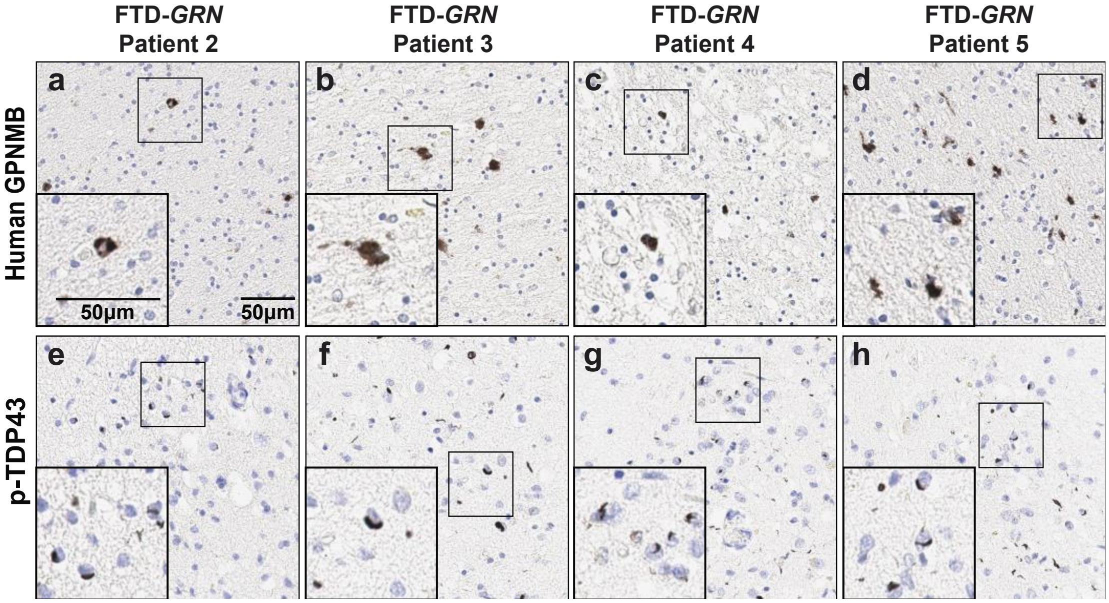

Supplementary Fig. S7 a-d GPNMB immunostaining was performed on frontal lobe tissue sections from 4 FTD-*GRN* patients. **e-h** Immunostaining for p-TDP 43 was stained on adjacent sections from identical samples as marker of FTLD pathology. Scale bars (50 µm) labeled in images.
